# Hypoxia-inducible lipid droplet-associated induces DGAT1 and promotes lipid storage in hepatocytes

**DOI:** 10.1016/j.molmet.2021.101168

**Published:** 2021-01-16

**Authors:** Montserrat A. de la Rosa Rodriguez, Lei Deng, Anne Gemmink, Michel van Weeghel, Marie Louise Aoun, Christina Warnecke, Rajat Singh, Jan Willem Borst, Sander Kersten

**Affiliations:** 1 Nutrition, Metabolism, and Genomics Group, Division of Human Nutrition and Health, Wageningen University, Stippeneng 4, Wageningen, 6708 WE, the Netherlands; 2 Department of Nutrition and Movement Sciences, Maastricht University Medical Center+, Maastricht, 6200 MD, the Netherlands; 3 Laboratory Genetic Metabolic Diseases, Amsterdam UMC, University of Amsterdam, Meibergdreef 9, Amsterdam, 1105 AZ, the Netherlands; 4 Department of Medicine, Albert Einstein College of Medicine, 1300 Morris Park Avenue, Forchheimer 505D, Bronx, NY, 10461, USA; 5 Department of Nephrology and Hypertension, University Hospital Erlangen, Friedrich-Alexander-University Erlangen-Nürnberg, Erlangen, Germany; 6 Laboratory of Biochemistry, Microspectroscopy Research Facility, Wageningen University, Stippeneng 4, Wageningen, 6708 WE, the Netherlands

**Keywords:** Lipid droplets, Liver, HILPDA, DGAT1, Fluorescence microscopy, Triglyceride synthesis

## Abstract

**Objective:**

Storage of triglycerides in lipid droplets is governed by a set of lipid droplet-associated proteins. One of these lipid droplet-associated proteins, hypoxia-inducible lipid droplet-associated (HILPDA), was found to impair lipid droplet breakdown in macrophages and cancer cells by inhibiting adipose triglyceride lipase. Here, we aimed to better characterize the role and mechanism of action of HILPDA in hepatocytes.

**Methods:**

We performed studies in HILPDA-deficient and HILPDA-overexpressing liver cells, liver slices, and mice. The functional role and physical interactions of HILPDA were investigated using a variety of biochemical and microscopic techniques, including real-time fluorescence live-cell imaging and Förster resonance energy transfer-fluorescence lifetime imaging microscopy (FRET-FLIM).

**Results:**

Levels of HILPDA were markedly induced by fatty acids in several hepatoma cell lines. Hepatocyte-specific deficiency of HILPDA in mice modestly but significantly reduced hepatic triglycerides in mice with non-alcoholic steatohepatitis. Similarly, deficiency of HILPDA in mouse liver slices and primary hepatocytes reduced lipid storage and accumulation of fluorescently-labeled fatty acids in lipid droplets, respectively, which was independent of adipose triglyceride lipase. Fluorescence microscopy showed that HILPDA partly colocalizes with lipid droplets and with the endoplasmic reticulum, is especially abundant in perinuclear areas, and mainly associates with newly added fatty acids. Real-time fluorescence live-cell imaging further revealed that HILPDA preferentially localizes to lipid droplets that are being remodeled. Overexpression of HILPDA in liver cells increased the activity of diacylglycerol acyltransferases (DGAT) and DGAT1 protein levels, concurrent with increased lipid storage. Confocal microscopy coupled to FRET-FLIM analysis demonstrated that HILPDA physically interacts with DGAT1 in living liver cells. The stimulatory effect of HILPDA on lipid storage via DGAT1 was corroborated in adipocytes.

**Conclusions:**

Our data indicate that HILPDA physically interacts with DGAT1 and increases DGAT activity. Our findings suggest a novel regulatory mechanism by which fatty acids promote triglyceride synthesis and storage.

## Highlights

• HILPDA expression is induced by fatty acids in hepatoma cells.
• HILPDA deficiency modestly decreases liver triglyceride storage in mice with NASH.
• HILPDA preferentially associates with newly synthesized lipid droplets and active lipid droplets.
• HILPDA promotes lipid storage at least in part independently of ATGL.
• HILPDA physically interacts and induces DGAT1.

## Graphical abstract

**Figure:**
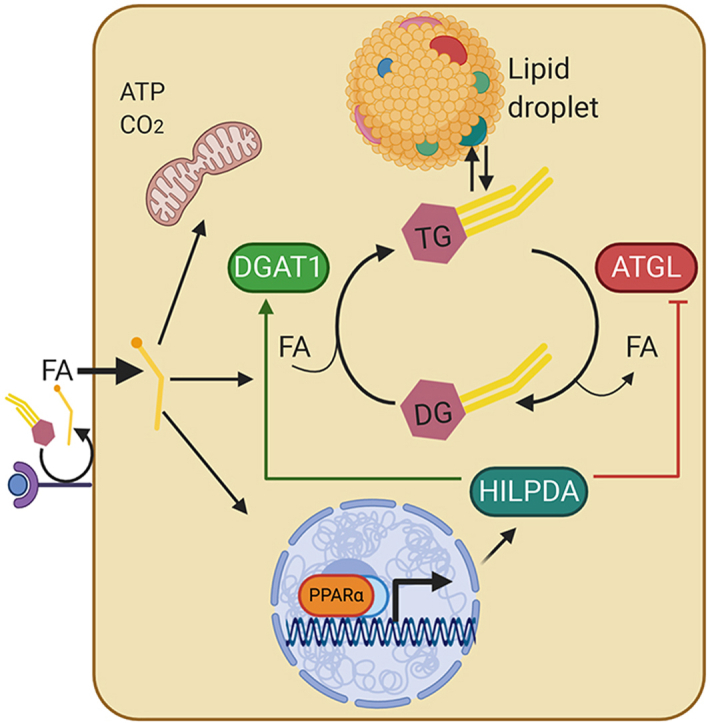

## 1 Introduction

Fatty acids are an important fuel for many cell types. When the supply of fatty acids exceeds the demand for oxidation, excess fatty acids can be stockpiled by converting them to triglycerides. Triglycerides are synthesized in the endoplasmic reticulum (ER) and are stored in specialized organelles called lipid droplets (LD) [1]. With the exception of adipocytes, most cell types have tiny LD that collectively only take up a very small portion of the total cell volume. However, in certain pathological conditions, LD may become enlarged and occupy considerable cell volume, potentially interfering with important cellular functions [2].

The liver plays a central role in the regulation of lipid metabolism. Under conditions of obesity and insulin resistance, storage of lipids in the liver is often elevated [3]. A chronic increase in intra-hepatic fat is referred to as steatosis and is a key feature of non-alcoholic fatty liver disease (NAFLD) [4]. In many high-income countries, NAFLD has become the most common liver disorder and is a growing clinical concern [4].

Fatty acids in hepatocytes can originate from several sources: from circulating triglycerides in chylomicron remnants and very low-density lipoprotein remnants taken up by the liver, from endogenous synthesis (de novo lipogenesis), and from circulating non-esterified fatty acids released by adipose tissue [5]. A portion of the incoming fatty acids is oxidized to provide energy to hepatocytes and, depending on nutritional status, converted into ketone bodies. The remainder is esterified into triglycerides, part of which is incorporated in and secreted as very low-density lipoproteins, and part of which is stored in LD. Accordingly, excess storage of lipids in the liver can be due to changes in several metabolic pathways, including defective fatty acid oxidation, enhanced lipogenesis, impaired triglyceride secretion, and increased uptake of fatty acids from the circulation [6].

LD are dynamic organelles that can rapidly expand and shrink, driven by fluctuations in the rate of triglyceride synthesis and degradation [2]. The synthesis of triglycerides, their storage in LD, and the subsequent breakdown of triglycerides into fatty acids are governed by a complex set of enzymes and LD-associated proteins. According to proteomic profiling, the number of LD-associated proteins easily runs into the hundreds [[7], [8], [9], [10]]. LD-associated proteins encompass a large group of proteins that physically and functionally interact with LD. This group includes lipid synthesis and degradation enzymes, proteins involved in membrane trafficking, lipid signaling proteins, and proteins involved in protein degradation [10]. An important group of LD-associated proteins is the perilipin family that comprises PLIN1-PLIN5 [11]. Other known LD-associated proteins include CIDEA, CIDEB, CIDEC, FITM1, FITM2, G0S2, and ABHD5 [[12], [13], [14]].

A relatively poorly characterized LD-associated protein is HILPDA. The first identification of HILPDA as LD-associated protein was in HeLa cells. In these cells, HILPDA overexpression increased intracellular lipid accumulation [15]. HILPDA raised our attention when trying to identify novel target genes of the transcription factors PPARα and PPARγ in hepatocytes and adipocytes, respectively, and when screening for novel genes induced by fatty acids in macrophages [[16], [17], [18]]. In mouse liver, HILPDA overexpression via adeno-associated viral (AAV) delivery raised intrahepatic triglyceride levels by approximately 4-fold, at least partly by suppressing the secretion of triglycerides in very low-density lipoproteins [17]. Consistent with these data, deficiency of HILPDA in cultured hepatocytes lowered hepatic lipid accumulation, which was explained by a combination of decreased fatty acid uptake, increased fatty acid beta-oxidation, and increased triglyceride lipolysis [19]. Somewhat surprisingly, hepatocyte-specific HILPDA deficiency did not influence liver triglyceride content in mice chronically fed a high-fat diet [19].

Recently, we and others found that HILPDA is able to bind to the intracellular triglyceride hydrolase ATGL and inhibits ATGL-mediated triglyceride hydrolysis [18,[20], [21], [22]]. Studies in HILPDA-deficient macrophages and cancer cells have firmly established the physiological relevance of ATGL inhibition by HILPDA [18,[20], [21], [22]]. Currently, very little is known about the molecular mechanism of action of HILPDA in hepatocytes. Accordingly, the present study was aimed at better characterizing the molecular role of HILPDA in hepatocytes.

## 2 Methods

### 2.1 Mice experiments

*Hilpda*^flox/flox^ mice (Jackson Laboratories, Bar Harbor, ME; Hilpdatm1.1Nat, #017360) were acquired and crossed with C57Bl/6J mice for at least 5 generations. Thereafter, the *Hilpda*^flox/flox^ mice were crossed with Albumin-Cre transgenic mice (Jackson Laboratories, Bar Harbor, ME; B6.Cg-Speer6-ps1^Tg(Alb-cre)21Mgn^/J, #003574) or Adiponectin-Cre transgenic mice (Jackson Laboratories, Bar Harbor, ME; B6.FVB-Tg (Adipoq-cre)1Evdr/J, # 028020) to generate mice with hepatocyte-specific Cre-mediated deletion of *Hilpda* (*Hilpda*^Δliver^) or adipocyte-specific Cre-mediated deletion of *Hilpda* (*Hilpda*^ΔADIPO^). Mice were group housed under normal light–dark cycles in temperature- and humidity-controlled specific pathogen-free conditions. Mice had ad libitum access to regular chow and water. A power calculation was performed based on fasting glucose level, assuming a standard deviation of 2.5 mM, using a power of 0.8, a significance level of 0.05, and an effect size of 3 mM. Using an online statistics tool from the University of British Columbia (https://www.stat.ubc.ca/∼rollin/stats/ssize/n2.html), the sample size was calculated as n = 11 mice per group. To allow compensation for potential loss of mice during the study, n = 12 mice were included per group.

Male *Hilpda*^Δliver^ mice aged 3–4 months and their *Hilpda*^flox/flox^ littermates were given a semi-purified low-fat diet (10% kcal fat, A08051501) or high-fat diet lacking choline and methionine (45% kcal fat, A06071309) (Research Diets, Inc. New Brunswick, NJ). During the dietary intervention, the mice were housed individually. After 11 weeks, mice were euthanized in the ad libitum fed state between 8:15 h and 10.00 h. The number of mice per group was 12.

Prior to euthanasia, mice were anesthetized with isoflurane, and blood was collected via orbital puncture in tubes containing ethylenediaminetetraacetic acid (EDTA, Sarstedt, Nümbrecht, Germany). Immediately thereafter, mice were euthanized by cervical dislocation, after which tissues were excised, weighed, and frozen in liquid nitrogen or prepared for histology. Frozen samples were stored at −80 °C. Liver tissue was fixed in 4% formaldehyde solution in phosphate-buffered saline (PBS). All animal experiments were approved by the local animal welfare committee of Wageningen University (AVD104002015236, 2016.W-0093.007 and 2016.W-0093.017). The experimenter was blinded to group assignments during all analyses.

### 2.2 Plasma measurements

Blood collected in EDTA tubes (Sarstedt, Numbrecht, Germany) was spun down for 10 min at 2,000 g at 4 °C. Plasma was aliquoted and stored at −80 °C until further measurements. The plasma concentration of various metabolites was determined using specialized kits: cholesterol (LiquiColor, Human GmbH, Wiesbaden, Germany), triglycerides (LiquiColor), glucose (LiquiColor), NEFA (NEFA-HR set R1, R2 and standard, WAKO Diagnostics, Instruchemie, Delfzijl, The Netherlands), Alanine Transaminase Activity Assay Kit (ab105134, Abcam, Cambridge, UK), following the manufacturer's instructions.

### 2.3 Liver triglycerides

Two-percent liver homogenates were prepared in buffer (10 mM Tris, 2 mM EDTA and 0.25 M sucrose, pH 7.5) using a Tissue Lyser II (Qiagen, Hilden, Germany). Liver triglyceride content was quantified using a Triglyceride LiquiColor mono kit from HUMAN Diagnostics (Wiesbaden, Germany) according to the manufacturer's instructions.

### 2.4 Cell treatments and gene expression

Human HepG2, mouse Hepa 1–6 and rat Fao hepatoma cells at 75% confluency were incubated with a mixture of oleate and palmitate (ratio 2:1, total concentration 1.2 mM) coupled to FA-free BSA (Roche Applied Sciences). All fatty acid stocks were initially reconstituted in absolute ethanol. Sub-stocks of fatty acids at 25 mM were prepared in filter-sterilized KOH at 70 mM. Fatty acids were diluted in Dulbecco's modified Eagle's medium (DMEM) containing 3% FA-free BSA to obtain the desired final concentrations. After treatment, cells were washed with ice-cold PBS (Lonza) and stored at −20 °C for further analysis.

Total RNA was isolated using TRIzol® Reagent (Invitrogen, ThermoFisher Scientific). cDNA was synthesized from 500 ng of RNA using the iScript cDNA kit (Bio-Rad Laboratories, Hercules, CA, USA) according to the manufacturer's instructions. Real-time polymerase chain reaction (RT-PCR) was performed with the CFX96 or CFX384 Touch™ Real-Time detection system (Bio-Rad Laboratories), using a SensiMix™ (BioLine, London, UK) protocol for SYBR green reactions. Mouse 36b4 expression was used for normalization.

### 2.5 Liver slices

Precision-cut liver slices were prepared from *Hilpda*^Δliver^ and *Hilpda*^flox/flox^ mice as described previously [23]. Briefly, 5 mm cylindrical liver cores were obtained with a surgical biopsy punch and sectioned to 200-μm slices using a Krumdieck tissue slicer (Alabama Research and Development, Munford, AL, USA) filled with carbonated Krebs-Henseleit buffer (pH 7.4, supplemented with 25 mM glucose). At this time point, some liver slices were snap-frozen in liquid nitrogen for RNA isolation. The rest were incubated in William's E Medium (Gibco, Paisley, Scotland) supplemented with penicillin/streptomycin in 6-well plates at 37 °C/5% CO_2_/80% O_2_ under continuous shaking (70 rpm). Three liver slices were incubated per well. After 1 h, medium was replaced with either fresh William's E Medium 1% BSA in the presence or absence of a mix of 0.8 mM of oleic acid and 0.02 mM of BODIPY FL C12 (ThermoFisher Scientific, Breda, Netherlands) for imaging or William's E Medium 1% BSA in the presence or absence of a mixture of oleate and palmitate (ratio 2:1, total concentration 0.8 mM) for RNA and protein isolation. After overnight incubation, liver slices were snap-frozen in liquid nitrogen and stored at −80 °C for RNA and protein isolation. Alternatively, liver slices were fixed for 1 h in 3.7% formaldehyde, transferred into an 8-well removable chamber (ibidi, GmbH, Martinsried, Germany), and coated with VECTASHIELD. Slices were imaged on a Leica TCS SP8 X confocal. BODIPY FL C12 was excited at 488 nm and detected using HyD in a spectral window of 505–550 nm.

### 2.6 Primary hepatocytes

Buffers: Hanks: 112 mM NaCl, 5.4 mM KCl, 0.9 mM KH_2_PO_4_, 0.7 mM Na_2_HPO_4_.12H_2_O. Hanks I: Hanks supplemented with 25 mM NaHCO_3_, 10 mM d-glucose, 0.5 mM EGTA at pH 7.42. Hanks II: same as Hanks I with the addition of 5 mM CaCl_2_. Krebs: 25 mM NaHCO_3_, 10 mM d-glucose, 10 mM Hepes. Hepatocyte culture medium: Williams E without phenol red (Fisher) supplemented with Primary hepatocyte maintenance supplement (Fisher). All buffers were saturated with carbogen before use.

Primary hepatocytes were prepared from *Hilpda*^Δliver^ and *Hilpda*^flox/flox^ mice. Briefly, mice were anesthetized with isoflurane. Livers were infused through the portal vein with Hanks buffer I for 10 min and with 100 mL of Hanks buffer II. Next, livers were infused with 200 mL of Liver Digest Medium (Fisher). Livers were excised and washed in Krebs Buffer. Primary hepatocytes were passed through a 100-μm mesh and centrifuged at 450 rpm for 4 min at 4 °C. The supernatant was discarded, and cells were washed again in cold Krebs medium. The supernatant was discarded, and cells were resuspended in hepatocyte culture medium with 5% fetal calf serum (FCS) and seeded on collagen-coated 8-well μ-slide glass bottom slides (Ibidi, Martinsried, Germany). After 2 h, medium was refreshed with hepatocyte culture medium with 10% FCS and left overnight. The next day, cells were treated with 20 μM of Atglistatin (Sigma–Aldrich) or dimethyl sulfoxide (DMSO) control and left overnight. The next morning, treatments were refreshed for 2 h before adding a mixture of oleate and palmitate (ratio 2:1, total concentration 0.8 mM). Cells were lipid loaded for 6 h and then fixed for 20 min in 3.7% paraformaldehyde (PFA). Lipid droplets were stained with 3 μg/mL of BODIPY® 493/503 and mounted with VECTASHIELD for imaging. Cells were imaged on a Leica TCS SP8 X confocal. BODIPY was excited at 488 nm and detected using HyD in a spectral window of 505–550 nm. Images were acquired at 1024 × 1024 pixels with pinhole set at 1 airy unit (AU), and pixel saturation was avoided. Images were processed and analyzed with Fiji. Briefly, images were converted to binary images, watershed, and LD size and number were measured with a particle analysis set of 0.07 μm^2^-infinity.

### 2.7 Primary adipocytes

Primary adipocytes were differentiated from the stromal vascular fraction, which was obtained from inguinal white adipose tissue of *Hilpda*^ΔADIPO^ and *Hilpda*^flox/flox^ mice. Briefly, dissected adipose tissue depots were kept and cleaned in ice-cooled transport medium (DMEM plus 1% fatty acid–free BSA (Sigma–Aldrich)). Cleaned adipose tissue samples were minced into small pieces and incubated with collagenase solution (DMEM, 3.2 mM CaCl_2_, 15 mM HEPES, 0.5% BSA, 10% FCS, and 1.5 mg/mL collagenase type II (Sigma–Aldrich; C6885)) at 37 °C for 30 min. The digested tissue suspensions were then filtered using a 100-mm cell strainer and centrifuged at 300 g for 10 min at room temperature. The pellet stromal vascular fraction (SVF) were resuspended and grown in cell culture flasks until around 90% confluency. Cells were seeded in the culture plate with a density of 15,000 cells/cm^2^ in DMEM, 10% FCS, and 1% penicillin/streptomycin. Two to 3 days post seeding (at full confluency), differentiation was started by supplementing with 0.5 mM of 3-isobutyl-1-methylxanthine (Sigma–Aldrich; I5879), 1 μM of dexamethasone (Sigma–Aldrich; D4902), 7 μg/mL of human insulin (Sigma–Aldrich; I2643), and 1 μM of rosiglistazone (Sigma–Aldrich; R2408). After 3 days of stimulation, cells were further cultured in insulin medium (DMEM containing 7 μg/mL human insulin) for another 3 days followed by normal growth medium (DMEM, 10% FCS and 1% penicillin/streptomycin). Gene expression of *Fabp4*, *Slc2a4*, *Adipoq*, *Pnpla2,* and *G0S2* was used as an indicator of cell differentiation.

For confocal imaging, the SVF was seeded and differentiated in 8-well μ-slide glass bottom slides coated with collagen. Differentiated adipocytes were stained with 2 μg/mL of BODIPY® 493/503 and mounted with VECTASHIELD after fixation with 3.7% paraformaldehyde. Imaging settings were as described previously. The size and fluorescent intensity of BODIPY-stained LD were analyzed using ImageJ (LOCI, University of Wisconsin). The size was measured from 0 μm^2^-infinity. To measure the release of non-esterified free fatty acids into the cell culture medium, adipocytes were starved with DMEM supplied with 0.5% BSA for 2 h, and medium was then collected and analyzed using a kit (Instruchemie, Delfzijl, the Netherlands) following the manufacturer's instruction. Adipocytes were treated with the ATGL inhibitor Atglistatin (50 μM) or the DGAT1 inhibitor T863 (20 μM) (Sigma–Aldrich) during starvation.

### 2.8 TG quantification in HepG2 cells

HepG2 cells were seeded in 24-well plates. The next day, cells were transduced with adenovirus-GFP (AV-*Gfp*) or adenovirus-mHilpda (AV-*Hilpda*) at 5 × 10^6^ IFU/mL media in DMEM (Lonza, Verviers, Belgium) supplemented with 10% fetal calf serum (Lonza) and 1% penicillin/streptomycin (Lonza), hereafter referred to as complete DMEM, and left overnight. Recombinant adenoviruses were generated by cloning *GFP* or mouse *Hilpda* cDNA in human adenovirus type5 (dE1/E3). Expression was regulated by CMV promoter. Viruses were produced and titrated by Vector Biolabs (Philadelphia, PA, USA). Cells were then incubated with DMEM 3% BSA and a mixture of oleate and palmitate (ratio 2:1, total concentration 1 mM) for 3 h. Cells were washed twice with PBS and frozen in 25 mM of Tris/HCl and 1 mM of EDTA, pH 7.5. TG quantification plates were thawed, and a mixture of 4:1 tertiary butanol:methanol was added to cells, followed by incubation of 10 min on a shaking platform. Plates were left to evaporate on a hot plate at 50 °C. Next, 300 μL of Triglyceride LiquiColor reagent (Human Diagnostics, Wiesbaden, Germany) was added to the cells and incubated for 10 min while shaking. One hundred microliters were transferred to a 96-well plate, and absorption was measured at 492 nm. A calibration curve of a standard solution was used to determine the TG content of the cells.

### 2.9 LD count

HepG2 and Hepa1-6 cells were plated on collagen-coated 8-well μ-slide glass bottom slides (Ibidi, Martinsried, Germany). The next day, cells were transduced with AV-*Hilpda* in complete DMEM at 5 × 10^6^ IFU/mL media and left overnight. HepG2 cells were incubated with a mixture of oleate and palmitate (ratio 2:1, total concentration 0.8 mM) for 8 h to promote LD formation. Hepa 1–6 cells were incubated with 1 mM of oleate:palmitate for 24 h. Cells were washed with PBS, fixed for 15 min with 3.7% formaldehyde, stained with 3 μg/mL of BODIPY® 493/503 and Hoechst for 45 min, and mounted with VECTASHIELD-H (Vector Laboratories). Cells were imaged on a Leica confocal TCS SP8 X system equipped with a 63 × 1.20 NA water-immersion objective lens. BODIPY® 493/503 was excited at 488 nm, and fluorescence emission was detected using internal Hybrid (HyD) in a spectral window of 505–578 nm. Images were acquired at 1,024 × 1,024 pixels, with pinhole set at 1 AU. Images were processed and analyzed with Fiji. Briefly, images were converted to binary, watershed, and LD size and number was measured with particle analysis set 0.07 μm^2^-infinity.

### 2.10 Western blot

Cell or tissue protein lysates were separated by sodium dodecyl sulfate-polyacrylamide gel electrophoresis (SDS-PAGE) on pre-cast 8–16% polyacrylamide gels and transferred onto nitrocellulose membranes using a Trans-Blot® Semi-Dry transfer cell (all purchased from Bio-Rad Laboratories), blocked in non-fat milk, and incubated overnight at 4 °C with primary antibody for HILPDA (1:750, Santa Cruz Biotechnology, sc-137518 or rabbit antisera against the C-terminal half (aa 37–64) of murine HILPDA generated by Pineda (Berlin, Germany) [24]), ACTIN (Cell Signaling Technology), ATGL (Santa Cruz Biotechnology), DGAT1 (Santa Cruz Biotechnology, sc-271934) or HSP90 (1:5,000, Cell Signaling Technology, #4874). Membranes were incubated with a secondary antibody (Anti-rabbit IgG, HRP-linked Antibody, 7074, Cell Signaling Technology) and developed using Clarity ECL substrate (Bio-Rad Laboratories). Images were captured with the ChemiDoc MP system (Bio-Rad Laboratories).

### 2.11 Plasmid constructs

Plasmids for *Plin2*, *Plin3*, *Gpat1*, *Gpat4*, *Dgat1*, *Dgat2,* and *Hilpda* were constructed by fusing the full-length mouse cDNA into pEGFP-N2 (Clonetech, Mountain View, California, USA) and substituting the EGFP sequence by the sequence of the fluorescent proteins (FP) mCherry, sYFP2 or mEGFP. Briefly, RNA from mouse WAT or liver was reverse transcribed with a First Strand cDNA synthesis kit (Thermo Fisher Scientific) and amplified with Phusion High-fidelity DNA Polymerase (Thermo Fisher Scientific) using gene-specific primers. The PCR products were cloned into pEGFP-N2 vector using the XhoI and KpnI-HF or NheI and BamHI (New England Biolabs Inc.) restriction enzyme sites. Afterwards, MAX Efficiency ® DH5α™ Competent Cells (Invitrogen) were transformed by heat-shock and grown in Luria–Bertani (LB) agar plates with kanamycin (Sigma–Aldrich). The vector was isolated using a Qiagen plasmid maxi kit (Qiagen) according to the manufacturer's instructions. The EGFP sequence was then excised from the pEGFP-N2 parent vector by enzyme digestion with KpnI-HF and NotI-HF. The vector was gel-purified with QIAquick Gel Extraction Kit (Qiagen), and the fragments of mCherry, sYFP2 or mEGFP were ligated into KpnI and NotI restriction enzyme sites using T4 DNA ligase (Thermo Scientific). For plasmids of mGPAT1 and mGPAT4 the original pEGFP-N2 plasmid was used.

### 2.12 Stimulated emission depletion (STED) microscopy

HepG2 cells were plated on collagen-coated 8-well micro-slide glass bottom slides (Ibidi, Martinsried, Germany). The next day, cells were transfected with 750 ng of *Hilpda*_sYFP2 complexed to polyethylenimine (PEI) (Polyscience Inc., PA, USA) in serum-free medium. After 6 h, the transfection medium was changed to DMEM 1% FA-free BSA with 0.8 mM OA and 15 μM BODIPY C12 558/568. Cells were fixed after 18 h of lipid loading for 20 min in 3.7% PFA. Images were acquired on a Leica TCS SP8 STED microscope. A 100 × 1.4 N A. oil immersion objective was used in combination with a 5× optical zoom, resulting in a pixel size of 23 × 23 nm. The pinhole was set at 0.9 AU and imaging speed at 700 Hz. For excitation of the fluorescent probes, a white light laser line was used. HILPDA-sYFP2 and BODIPY-558 were excited at 470 nm and 558 nm, respectively, and fluorescence emission was detected using HyD in a spectral window of 480–540 nm and 570–650 nm, respectively. The HILPDA-sYFP2 emission was partly depleted with the 592 depletion laser set at a 40% laser intensity with a power output of 1.3530 W. For both fluorophores, the gating was set at 0.3–6.5 ns. Images were corrected for chromatic aberration and deconvolved using the Deconvolution Express modus in Huygens Professional Software (Scientific Volume Imaging B.V., Hilversum, the Netherlands).

### 2.13 HILPDA and fluorescently labeled fatty acid colocalization

HepG2 cells were plated on collagen-coated 8-well micro-slide glass bottom slides (Ibidi, Martinsried, Germany). The next day, cells were transfected with Hilpda_mTurquoise2 plasmid complexed to PEI in serum-free DMEM. After 6 h, the medium was replaced by complete DMEM and left overnight. Cells were then incubated for 16 h with 0.6 mM of oleate and 15 μM of BODIPY C12 558/568 and the next day for 20 min with QBT fatty acid uptake solution, which uses a BODIPY FL ®-dodecanoic acid fluorescent fatty acid analog (BODIPY FL C12), prepared according to the manufacturer's protocol (Molecular Devices, California, USA). Cells were washed with PBS, fixed with 3.7% formaldehyde for 30 min, and mounted with VECTASHIELD (Molecular Devices, California, USA). Imaging was performed on a Leica TCS SP8 X system equipped with a 63×1.20 NA water-immersion objective lens. Images were acquired sequentially as 1,024 × 1,024 pixel scans with pinhole set at 1 AU. mTurquoise2 was excited at 440 nm and fluorescence emission was detected using internal HyD in a spectral window of 450–480 nm. BODIPY C12 558/568 was excited at 561 nm and fluorescence emission was detected using internal Hybrid (HyD) in a spectral window of 570–620 nm. BODIPY FL C12 was excited at 488 nm and fluorescence emission was detected using internal HyD in a spectral window of 505–558 nm. During image acquisition, fluorescence bleed-through and pixel saturation were avoided. All images were deconvolved using Deconvolution Express modus with Huygens Essential version 18.10 (http://svi.nl, The Netherlands). Further images were processed with ImageJ. Briefly, channels were split, the entire cell was selected as a ROI, colocalization threshold was used to obtain colocalized pixels image, and Mander's colocalization (overlap) coefficient and Pearson correlation coefficient were measured using Coloc2 plugin.

### 2.14 2D Time Lapse

HepG2 cells were seeded on 15-μm 8-well glass bottom slides (Ibidi, Martinsried, Germany) and grown overnight before transfection. Cells were transfected with 800 ng of mHilpda_mCherry plasmid complexed to polyethylenimine (PEI) (Polyscience Inc., PA, USA) in serum-free DMEM. After 6 h, the medium was replaced by complete medium. The next day, cells were starved for 1 h with HBSS 0.2% FA-free BSA. Medium was then replaced with QBT fatty acid uptake assay kit, and after 4 h of incubation, cells were imaged on a Leica TCS SP8 X system equipped with a 63×1.20 NA water-immersion objective lens. Images were acquired sequentially using 512 × 512 pixels, and a total of 491 frames were acquired with a frame interval of ±5 s. All images were deconvolved using Deconvolution Express modus with Huygens Essential version 18.10 (Scientific Volume Imaging, The Netherlands, http://svi.nl). Further images were processed with Fiji to assign different coloring LUTs for visualization.

### 2.15 Lipophagy assay

HepG2 cells were transduced with AV-*GFP* or AV-*Hilpda* in complete DMEM at 5 × 10^6^ IFU/mL media and left overnight. The next day, HepG2 cells were incubated with a mixture of 8 mM of oleate:palmitate (ratio 2:1) for 8 h to promote LD formation. Two hours before collection, cells were treated with a lysosomal inhibitor cocktail, 20 mM of ammonium chloride and 100 uM of leupeptin. Cells were then lysed in radioimmunoprecipitation assay (RIPA) buffer with protease and phosphatase inhibitors and centrifuged at 10,000 rpm for 10 min.

### 2.16 FRET-FLIM analysis

FRET is a process in which the excitation energy is transferred from a donor fluorophore to an acceptor chromophore in very close proximity (<10 nm). FRET determined using FLIM is independent of the protein concentration but very sensitive to the local microenvironment of the fluorophores. In FRET-FLIM, the fluorescence lifetime of the donor molecule is reduced in the presence of a nearby acceptor molecule because energy transfer to the acceptor introduces an additional relaxation path from the excited to the ground state of the donor (34).

HepG2 cells were cultivated in complete DMEM (at standard conditions (37 °C, 5% CO2, 95% humidified atmosphere). Cells were seeded on a rat tail collagen-coated (Ibidi, Martinsried, Germany) 15-μm 8-well glass bottom slides (Ibidi, Martinsried, Germany) and grown for 24 h before transfection. Transfections were performed with 800 ng of single or 1600 ng of mixed plasmid DNA complexed to polyethylenimine (PEI) (Polyscience Inc., PA, USA) in serum-free DMEM. After 5 h, the medium was replaced by serum-free DMEM supplemented with 1% fatty acid-free BSA (Roche Applied Sciences) and a mixture of oleate and palmitate (ratio 2:1, total concentration 0.8 mM) and left overnight. For imaging, medium was replaced with FluoroBrite DMEM supplemented with 1% BSA and 0.8 mM fatty acid mix.

Colocalization imaging was performed on a Leica TCS SP8 X system equipped with a 63×1.20 NA water-immersion objective lens. Images were acquired sequentially at 512 × 512 pixels with pinhole set at 1 AU. mEGFP was excited at 488 nm and fluorescence emission was detected using internal HyD in a spectral window of 505–550 nm mCherry was excited at 561 nm and detected using HyD in a spectral window of 580–650 nm. All images were deconvolved using Deconvolution Express modus with Huygens Essential version 18.10 (Scientific Volume Imaging, The Netherlands, http://svi.nl). Further images were process with Fiji. Briefly, brightness and contrast levels were adjusted, and images were merged.

Förster resonance energy transfer-Fluorescence lifetime imaging microscopy (FRET-FLIM) was performed on a Leica TCS SP8 X confocal microscope. Donor and acceptor (mEGFP and mCherry, respectively) molecules were excited using a 40 MHz tunable supercontinuum laser at 488 nm and 561 nm, respectively. Fluorescence emission was detected using HyD detectors with 100 ps time resolution and collected in a spectral window of 505–550 nm for the donor (mEGFP) and 580–650 nm for the acceptor (mCherry). The signal output from the HyD detector was coupled to an external time-correlated single-photon counting module (Becker&Hickl) for acquiring FLIM data. Typical images had 256 × 256 pixels (pixel size ± 300 nm), and the analog to digital converter (ADC) was set to 256 time channels and FLIM images were acquired by imaging for 120 s per image. From the time-resolved fluorescence intensity images, the fluorescence decay curves were calculated for each pixel and fitted with a double-exponential decay model using the SPCImage v7.1 software (Becker & Hickl). Fitting was performed without fixing any parameters. FRET-FLIM analysis provided fluorescence intensity as well as false-colored fluorescence lifetime images. The raw data were subjected to the following criteria to analyze and omit false positive negatives in the fluorescence lifetime scoring: minimum photon count per pixel of 1,000 photons, 2 component analysis, goodness of fit (χ2<2) and fluorescence lifetime range of 500–3,500 ps. For data analysis, we set pixel binning at 1 to have a sufficient number of photons per pixel required for accurate fluorescence lifetime analysis.

### 2.17 DGAT assay

Our protocol is a modification of the method described by McFie and Stone [25]. HepG2 cells were seeded in 6-well plates at a density of 4 × 10^5^ cells/well or in 60 × 15 mm round cell culture dishes at a cell density of 3.5 × 10^6^ cells/dish in DMEM supplemented with 10% fetal calf serum (FCS) and 1% penicillin/streptomycin. The next day, cells were transduced with AV-*Gfp* or AV-*Hilpda* at 5 × 10^6^ PFU/mL. The next day, the medium was changed to complete DMEM with inhibitors. Atglistatin (40 μM), DGAT1 inhibitor (A922500, 1 μM), and DGAT2 inhibitor (PF-06424439, 40 μM), or control and incubated overnight. Atglistatin is a specific high-affinity inhibitor of ATGL [26]. For the samples treated with Atglistatin, Atglistatin was added again the next morning 2 h prior to cell lysate isolation. Cells were detached with trypsin, washed, and resuspended in 100 μL of 50 mM Tris–HCl (pH 7.6)/250 mM sucrose buffer supplemented with protease inhibitors (Roche Diagnostics GmbH). Cells were disrupted by 20 passages through a 27-gauge needle. Prepared cell lysate samples were placed on a spinning wheel for 20 min at 4 °C. Cell debris was pelleted by centrifugation at 2,500 rpm for 5 min. The supernatant was transferred to a new tube and used for the assay. Protein concentration was determined using a Pierce BCA kit (Thermo Fisher Scientific). A master mix containing 20 μL of 1 M Tris–HCl (pH 7.6), 4 μL of 1 M MgCl_2_, 10 μL of 4 mM DOG (Sigma–Aldrich), 10 μL of 12.5 mg/mL BSA, 10 μL of 500 μM NBD-palmitoyl CoA (Avanti Polar Lipids), and 96 μL of water per reaction, was prepared. Volumes were scaled up proportionally to accommodate the desired number of reactions. In addition, inhibitors were added to the master mix during the fluorescence assay in the above described concentrations. The master mix was protected from direct light during the entire experiment by wrapping the glass test tubes in aluminum foil. Assays were performed in 13 × 100 mm glass KIMAX Test Tubes with Teflon Liner Caps (DWK Life Sciences, Kimble) in a final reaction volume of 250 μL. A master mix volume of 150 μL was aliquoted per test tube, and tubes were pre-incubated in a 37 °C water bath for 2 min. The reaction was started by adding 300 ug in 100 μL of protein sample and incubated at 37 °C in a shaking water bath (GFL Gesellschaft für Labortechnik mbH, Product No. 1086) for 30, 90, and/or 180 min with steady shaking at 60 rpm. For the inhibitor treatments, samples were incubated for 180 min. The reaction was terminated by adding 4 mL of CHCl_3_/methanol (2:1, v/v) and 800 μL of water mixed by vortex. After 1 h, the test tubes containing samples were re-vortexed and centrifuged at 3,000 rpm for 5 min to separate aqueous and organic phases. The upper aqueous phase was aspirated, and the organic phase dried under stream of nitrogen. To help the solvents evaporate faster, the test tubes were placed in a thermal block . Lipids were finally resuspended in 50 μL of CHCl_3_/methanol (2:1) and stored at −20 °C overnight. Samples were vortexed and re-centrifuged at 3,000 rpm for 2 min before being spotted on channelled 20 × 20 cm TLC (thin-layer chromatography) plates with pre-adsorbent silica gel HLF zone (Analtech). The TLC plates were developed in the solvent system containing hexane/ethyl ether/acetic acid (80:20:1, v/v/v). The plates were air-dried for 1 h before quantification of reaction products.

The newly synthesized NBD-TG was analyzed with a ChemiDocTM MP molecular imaging system (Bio-Rad Laboratories, Inc.), and fluorescence was quantified with Quantity One software 4.1 (Bio-Rad Laboratories, Inc.). The excitation and emission wavelengths of NBD are 465 nm and 535 nm, respectively. Extinction source UV trans-illumination and a standard emission filter, together with Application SYBER Green and Applied (UV Trans Orange) Flat Field, were used. Data are presented as arbitrary fluorescence intensity units.

### 2.18 Microarray analysis

Microarray analysis was performed on Hepa1-6 hepatoma cells incubated with different fatty acids. RNA was purified with RNeasy Minikit columns (Qiagen) and analyzed for quality with RNA 6000 Nano chips on the Agilent 2100 bioanalyzer (Agilent Technologies, Amsterdam, The Netherlands). One microgram of RNA was used for cDNA synthesis using the First Strand cDNA synthesis kit (Thermo Scientific). Purified RNA (100 ng) was labeled with the Ambion WT expression kit (Invitrogen) and hybridized to an Affymetrix Mouse Gene 1.1 ST array plate (Affymetrix, Santa Clara, CA). Hybridization, washing, and scanning were carried out on an Affymetrix GeneTitan platform. Scans of the Affymetrix arrays were processed using packages from the Bioconductor project. Arrays were normalized using the robust multi-array average method [27,28]. Probe sets were defined by assigning probes to unique gene identifiers, e.g., Entrez ID [29]. The total gene set (24,973 probe sets) was filtered to only include genes with mean signal > 20, yielding 10,379 genes. Microarray data were submitted to the Gene Expression Omnibus (accession number pending).

### 2.19 Statistical analysis

Details of statistical analyses are given in the figure legends. Statistical analyses were carried out using an unpaired Student's t test or two-way analysis of variance (ANOVA). A value of p < 0.05 was considered statistically significant.

## 3 Results

### 3.1 Hilpda expression is induced by fatty acids in hepatoma cells

**Figure 1:**
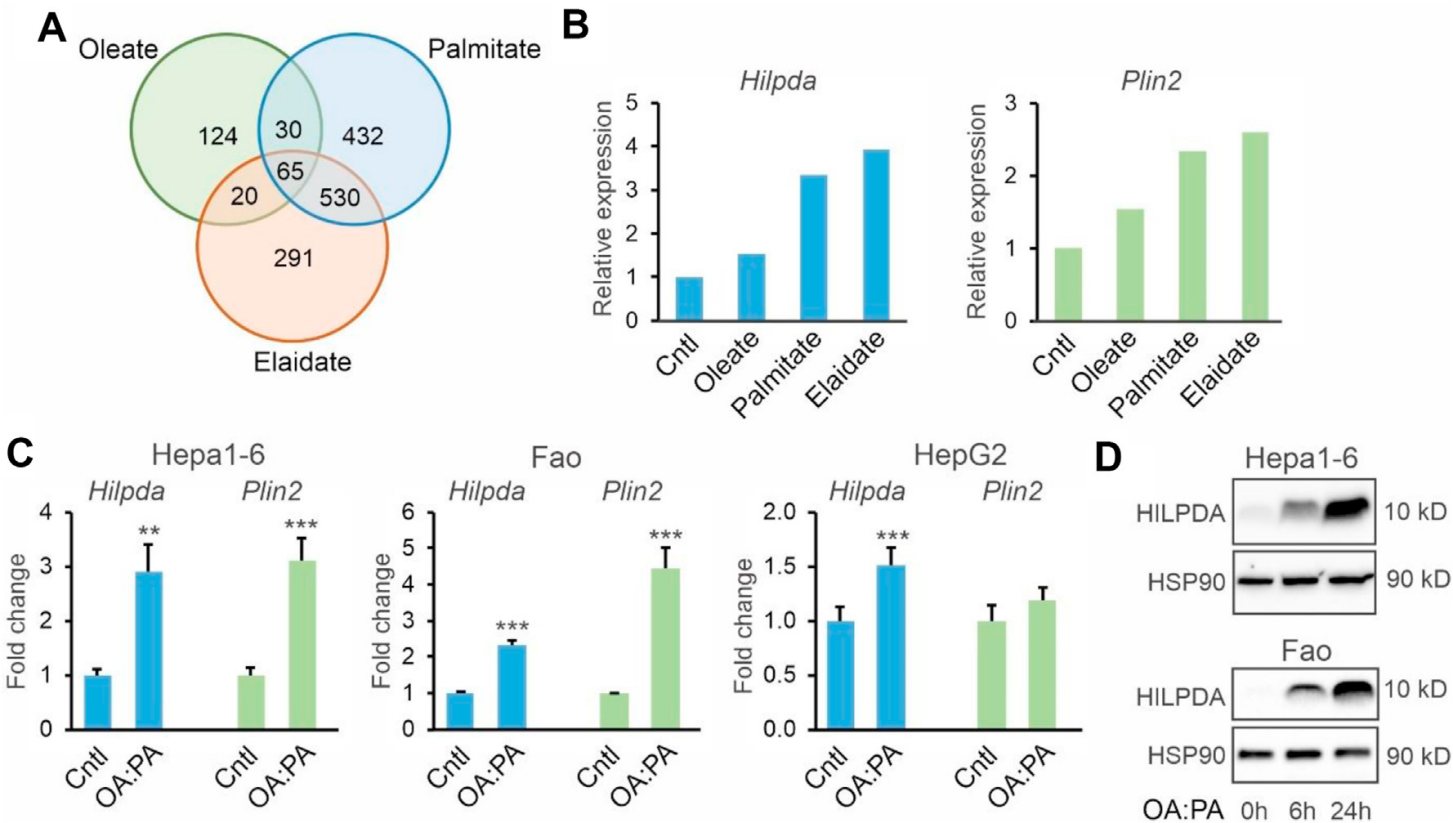
*Hilpda* expression is induced by fatty acids in various hepatoma cell lines. A) Venn diagram of upregulated genes (fold change>1.5) in murine Hepa1-6 hepatoma cells treated with different fatty acids (500 μM) for 6 h. B) Relative changes in *Hilpda* and *Plin2* mRNA in Hepa1-6 cells treated with different fatty acids (500 μM) for 6 h based on microarray analysis. C) Relative changes in *Hilpda* and *Plin2* mRNA in Hepa1-6, Fao and HepG2 hepatoma cells treated for 24 h with a 2:1 mixture of oleate and palmitate (OA:PA, total concentration 1.2 mM, n = 4–5 per condition). D) HILPDA protein levels in Hepa1-6 and Fao cells treated with a 2:1 mixture of oleate and palmitate (total concentration 1.2 mM) for different duration. Bar graphs are presented as mean ± SD. Asterisk indicates significantly different from control-treated cells according to Student's t-test; ∗∗P < 0.01; ∗∗∗P < 0.001.

To examine the regulation of *Hilpda* expression by fatty acids in liver cells, we treated mouse Hepa1-6 cells for 6 h with different types of fatty acids: cis-unsaturated (oleate), trans-unsaturated (elaidate), or saturated (palmitate). In addition to *Plin2*, *Hilpda* was one of the 65 genes that was induced at least 1.5-fold by palmitate (3.3-fold), oleate (1.5-fold), and elaidate (3.9-fold) (Figure 1A). The regulation of *Hilpda* strongly resembled the pattern observed for *Plin2* (Figure 1B). To further investigate the induction of *Hilpda* by fatty acids, different hepatoma cell types were treated with a mixture of oleate and palmitate. In mouse Hepa1-6, rat Fao, and human HepG2 cells, a mixture of oleate and palmitate significantly induced *Hilpda* mRNA, along with *Plin2* (Figure 1C). The upregulation of HILPDA by oleate and palmitate was confirmed at the protein level in Hepa1-6 cells and Fao cells (Figure 1D). These data indicate that *Hilpda* expression is induced by fatty acids in liver cells.

### 3.2 HILPDA deficiency modestly decreases liver triglyceride storage in mice with non-alcoholic steatohepatitis (NASH)

**Figure 2:**
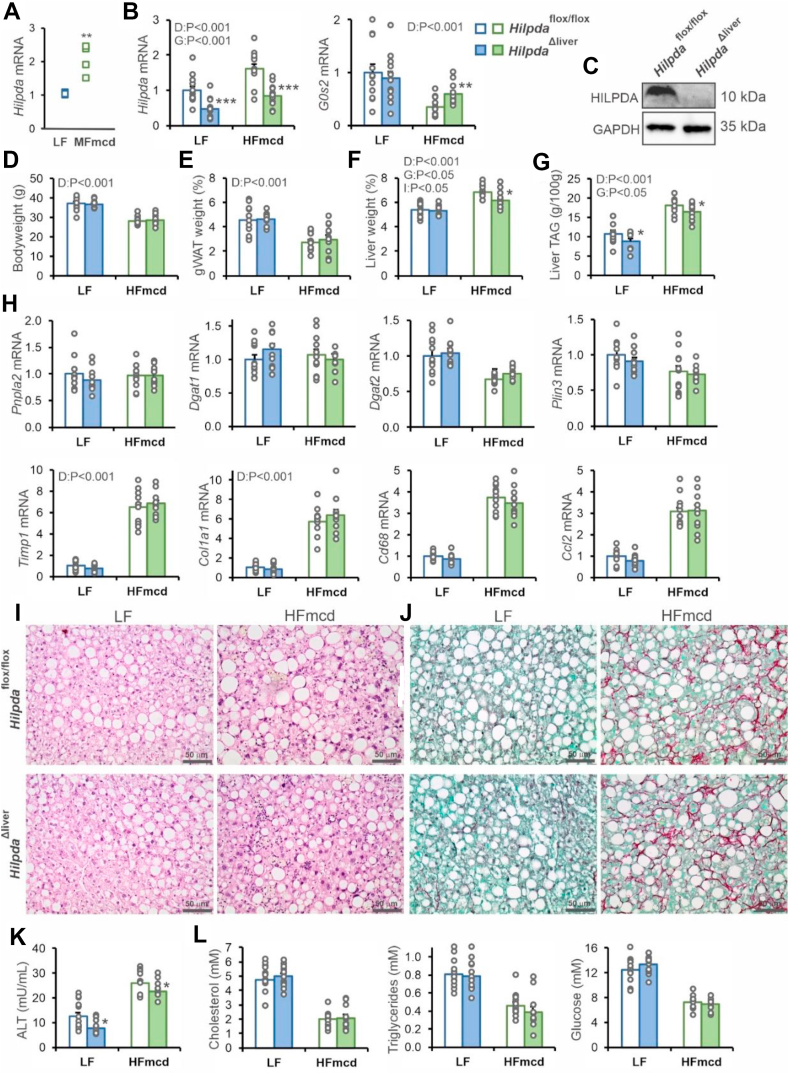
Effect of hepatocyte-specific HILPDA deficiency in mice with NASH. a) Upregulation of hepatic *Hilpda* mRNA by a methionine- and choline-deficient diet (GSE35961). N = 4 mice/group. B) *Hilpda* and *G0s2* mRNA levels in livers of *Hilpda*^Δliver^ and *Hilpda*^flox/flox^ mice fed a low-fat diet (LF) or high-fat diet deficient in methionine and choline (HFmcd). C) HILPDA protein levels in livers of *Hilpda*^Δliver^ and *Hilpda*^flox/flox^ mice fed a LF diet. D) Body weight. E) Gonadal adipose tissue weight. F) Liver weight. G) Liver triglyceride levels. H) mRNA levels of various LD-associated proteins, inflammatory markers, and fibrosis markers. I) H&E staining. J) Sirius Red staining. K) Plasma ALT levels. L) Plasma levels of various metabolites. Data are mean ± SEM; N = 12 mice/group. Asterisk indicates significantly different from *Hilpda*^flox/flox^ mice according to Student's t-test; ∗P < 0.05; ∗∗P < 0.01; ∗∗∗P < 0.001.

Previously, it was found that hepatocyte-specific deficiency of HILPDA reduced hepatic triglyceride levels under chow-fed conditions, although not after chronic high-fat feeding [19]. A possible reason for the inconsistent effect of HILPDA deficiency on liver triglycerides is the relatively low *Hilpda* expression in liver. To identify conditions in which deficiency of HILPDA may be expected to have a larger effect, we screened mouse liver transcriptome data for upregulation of *Hilpda*. Interestingly, hepatic *Hilpda* mRNA levels were increased during NASH caused by feeding mice a methionine- and choline-deficient diet (Figure 2A, based on GSE35961). Accordingly, we hypothesized that the effect of HILPDA deficiency may be more pronounced during NASH. Hepatocyte-specific HILPDA-deficient mice were generated by crossing *Hilpda*^flox/flox^ mice with mice expressing Cre-recombinase driven by the albumin promoter. To induce NASH, *Hilpda*^Δliver^ and *Hilpda*^flox/flox^ mice were fed a high-fat diet deficient in methionine and choline for 11 weeks (HFmcd), using a low-fat (LF) diet as a control. This HFmcd diet does not lead to weight loss and has been reported to be a better model for human NASH than the traditional methionine- and choline-deficient diet [30,31]. After 11 weeks, hepatic expression of *Hilpda* was significantly higher in mice fed the HFmcd than in mice fed the LF diet, and was significantly lower in *Hilpda*^Δliver^ than in *Hilpda*^flox/flox^ mice, which was accompanied by a modest compensatory increase in *G0s2* mRNA (Figure 2B). HILPDA protein levels were also markedly reduced in livers of *Hilpda*^Δliver^ compared to *Hilpda*^flox/flox^ mice (Figure 2C). Mice fed HFmcd were significantly lighter than the mice fed the LF diet, but no differences were observed between the genotypes (Figure 2D). Similarly, the weight of the epididymal fat pad was significantly lower in the mice fed HFmcd, but no differences were observed between *Hilpda*^Δliver^ and *Hilpda*^flox/flox^ mice (Figure 2E). Intriguingly, in mice fed HFmcd but not the LF diet, the weight of the liver was modestly but significantly lower in *Hilpda*^Δliver^ than in *Hilpda*^flox/flox^ mice (Figure 2F). Consistent with a stimulatory effect of HILPDA on liver fat, hepatic triglyceride levels were modestly but significantly lower in *Hilpda*^Δliver^ compared to *Hilpda*^flox/flox^ mice, both on the HFmcd and LF diet (Figure 2G).

HILPDA deficiency did not have any significant effect on mRNA levels of *Pnpla2*, *Dgat1*, *Dgat2*, and *Plin3* (Figure 2H). Also, despite a marked induction by HFmcd of the expression of macrophage/inflammatory markers *Cd68* and *Ccl2*, and fibrosis markers *Timp1* and *Col1a1*, no significant differences were observed between *Hilpda*^Δliver^ and *Hilpda*^flox/flox^ mice (Figure 2H). Histological analysis by hematoxylin and eosin (Figure 2I) and Sirius Red (Figure 2J) staining indicated that mice fed HFmcd exhibited classical features of NASH, including ballooning, inflammation, steatosis, and fibrosis. However, no clear and consistent differences were noticeable between *Hilpda*^Δliver^ and *Hilpda*^flox/flox^ mice. By contrast, and in agreement with the liver triglyceride levels, plasma alanine aminotransferase (ALT) levels were modestly but significantly lower in *Hilpda*^Δliver^ than in *Hilpda*^flox/flox^ mice, both on the HFmcd and LF diet (Figure 2K). Finally, plasma levels of cholesterol, triglycerides, and glucose were not significantly different between *Hilpda*^Δliver^ and *Hilpda*^flox/flox^ mice on either diet (Figure 2l). Overall, these data indicate that hepatocyte-specific HILPDA deficiency causes a modest but significant decrease in hepatic triglyceride storage, liver weight, and plasma ALT levels, without having a clear impact on features of NASH and various metabolic parameters.

### 3.3 HILPDA promotes lipid storage at least in part independently of ATGL

**Figure 3:**
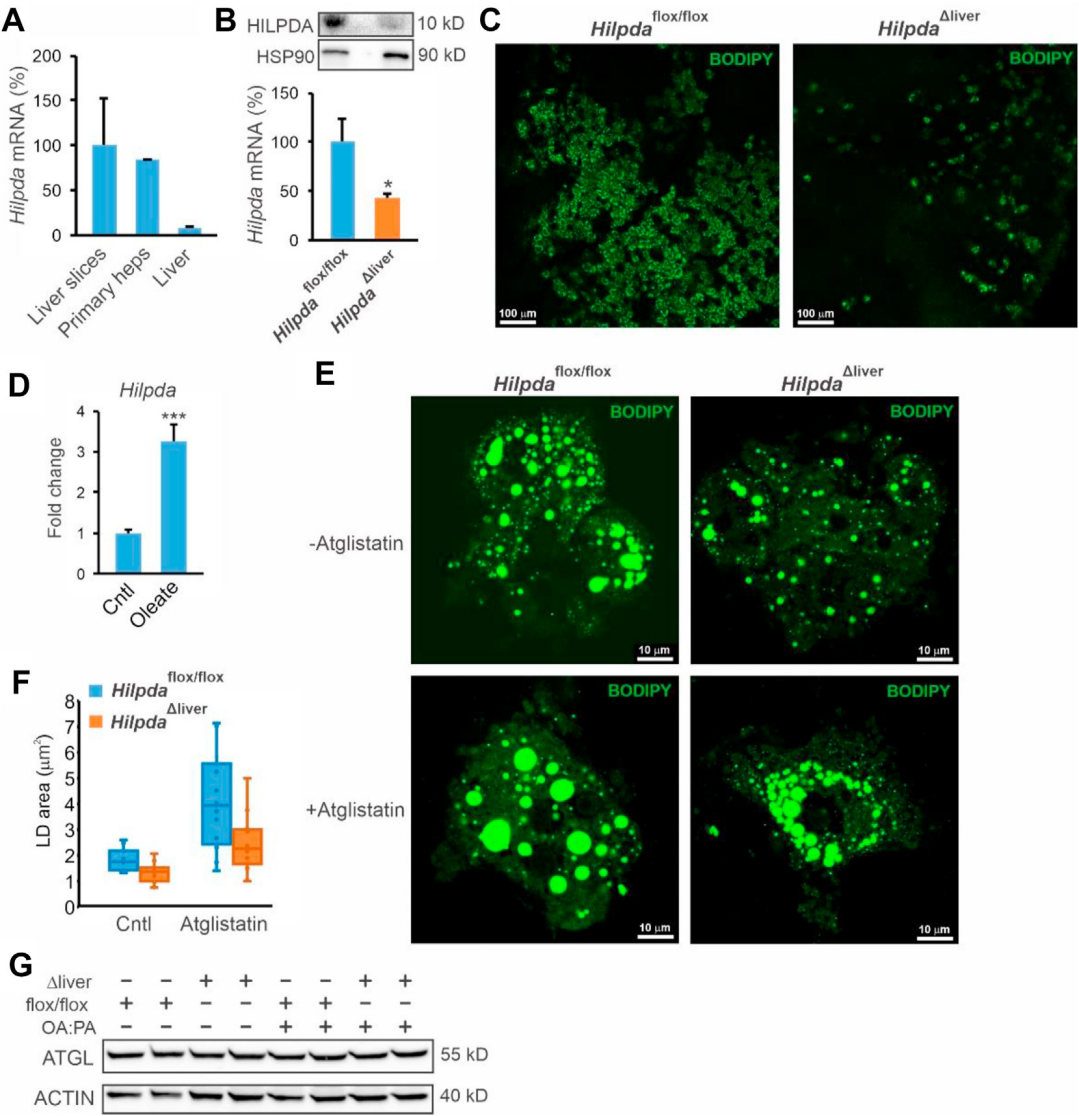
HILPDA stimulates lipid droplet formation partly independently of ATGL. a) Relative *Hilpda* mRNA levels in mouse precision cut liver slices, mouse primary hepactocytes, and mouse liver. B) HILPDA protein levels (top panel) and relative *Hilpda* mRNA levels (lower panel) in liver slices prepared from *Hilpda*^Δliver^ and *Hilpda*^flox/flox^ mice. Bar graphs are presented as mean ± SD. ∗ indicates P < 0.05 according to Student's t-test. C) Confocal microscopy of liver slices prepared from *Hilpda*^Δliver^ and *Hilpda*^flox/flox^ mice and incubated overnight with 800 μM oleate and 20 μM of BODIPY FL C12. λ_ex_: 488 nm, λ_em_: 550–595 nm. D) *Hilpda* mRNA expression in wildtype mouse primary hepatocytes treated with oleate (500 μM) for 24 h. ∗∗∗ indicates P < 0.001 according to Student's t-test. E) BODIPY 493/503 staining of primary hepatocytes prepared from *Hilpda*^Δliver^ and *Hilpda*^flox/flox^ mice and incubated overnight with 0.8 mM of oleate:palmitate mix (2:1) in the presence or absence of Atglistatin (20 μM). λ_ex_: 488 nm, λ_em_: 550–595 nm. F) Quantification of the lipid droplet area. The total number of lipid droplets analyzed per condition varied between 1,017 and 1,958. Two-way ANOVA revealed significant effects for Atglistatin (P < 0.001) and genotype (P < 0.001) but not for an interaction effect. G) ATGL protein levels in liver slices from *Hilpda*^Δliver^ and *Hilpda*^flox/flox^ mice and incubated overnight with 0.8 mM oleate:palmitate mix (2:1). Bar graphs and images are representative of at least two independent experiments.

To further study the functional role of HILPDA in liver cells, we used precision cut liver slices and primary hepatocytes. These model systems were chosen because they express very high levels of *Hilpda* compared to mouse liver (Figure 3A). Liver slices were prepared from *Hilpda*^Δliver^ and *Hilpda*^flox/flox^ mice and incubated overnight with a mixture of oleate and palmitate, as well as with BODIPY FL C12, followed by visualization of the stored fatty acids using fluorescence confocal microscopy. Levels of *Hilpda* mRNA were about 60% lower in *Hilpda*^Δliver^ than *Hilpda*^flox/flox^ liver slices, and HILPDA protein levels were also markedly reduced (Figure 3B). Consistent with a stimulatory effect of HILPDA on lipid storage, hepatocyte-specific deficiency of HILPDA led to a marked reduction in BODIPY FL accumulation in LD (Figure 3C).

We next moved to primary hepatocytes. In these cells, *Hilpda* mRNA was significantly induced by fatty acids (Figure 3D). To examine the effect of HILPDA deficiency on lipid storage, primary hepatocytes of *Hilpda*^Δliver^ and *Hilpda*^flox/flox^ mice were incubated overnight with a mixture of oleate and palmitate, followed by visualization of lipid storage by BODIPY 493/503 staining and fluorescence confocal microscopy. Again, consistent with a stimulatory effect of HILPDA on lipid storage, LD were considerably smaller in *Hilpda*^Δliver^ than *Hilpda*^flox/flox^ primary hepatocytes (Figure 3E). Quantification of the images revealed a significantly lower LD area in the *Hilpda*^Δliver^ than *Hilpda*^flox/flox^ hepatocytes (Figure 3F). Previously, we and others found that HILPDA inhibits ATGL [20,22]. To investigate whether the effect of loss of HILPDA on lipid storage in hepatocytes is mediated by hyperactivation of ATGL, we treated *Hilpda*^Δliver^ and *Hilpda*^flox/flox^ hepatocytes with the ATGL inhibitor Atglistatin. While Atglistatin and *Hilpda^flox/flox^* genotype significantly increased the LD area, no statistical interaction was observed between Atglistatin treatment and *Hilpda* genotype (Figure 3E,F), suggesting no functional interaction between ATGL and HILPDA. In contrast to HILPDA deficiency in macrophages [18,24], we also did not observe an increase in ATGL protein in HILPDA-deficient liver slices (Figure 3G). These data suggest that HILPDA promotes lipid storage at least partly via an ATGL-independent mechanism.

### 3.4 HILPDA preferentially associates with newly synthesized lipid droplets and active lipid droplets

To better understand the mechanism by which HILPDA promotes lipid storage in liver cells, we first investigated HILPDA localization. Previously, HILPDA protein was shown to partly localize to LD and to the LD-ER interface [15,16,19,24]. Cellular fractionation confirmed the association of HILPDA with LD in the mouse liver, which was reduced by fasting (Supplemental Fig. 1a). The fractionation of the liver was confirmed by immunoblot of marker genes, showing the pronounced activation of autophagy by fasting in the LD fraction (Supplemental Fig. 1b).

**Figure 4:**
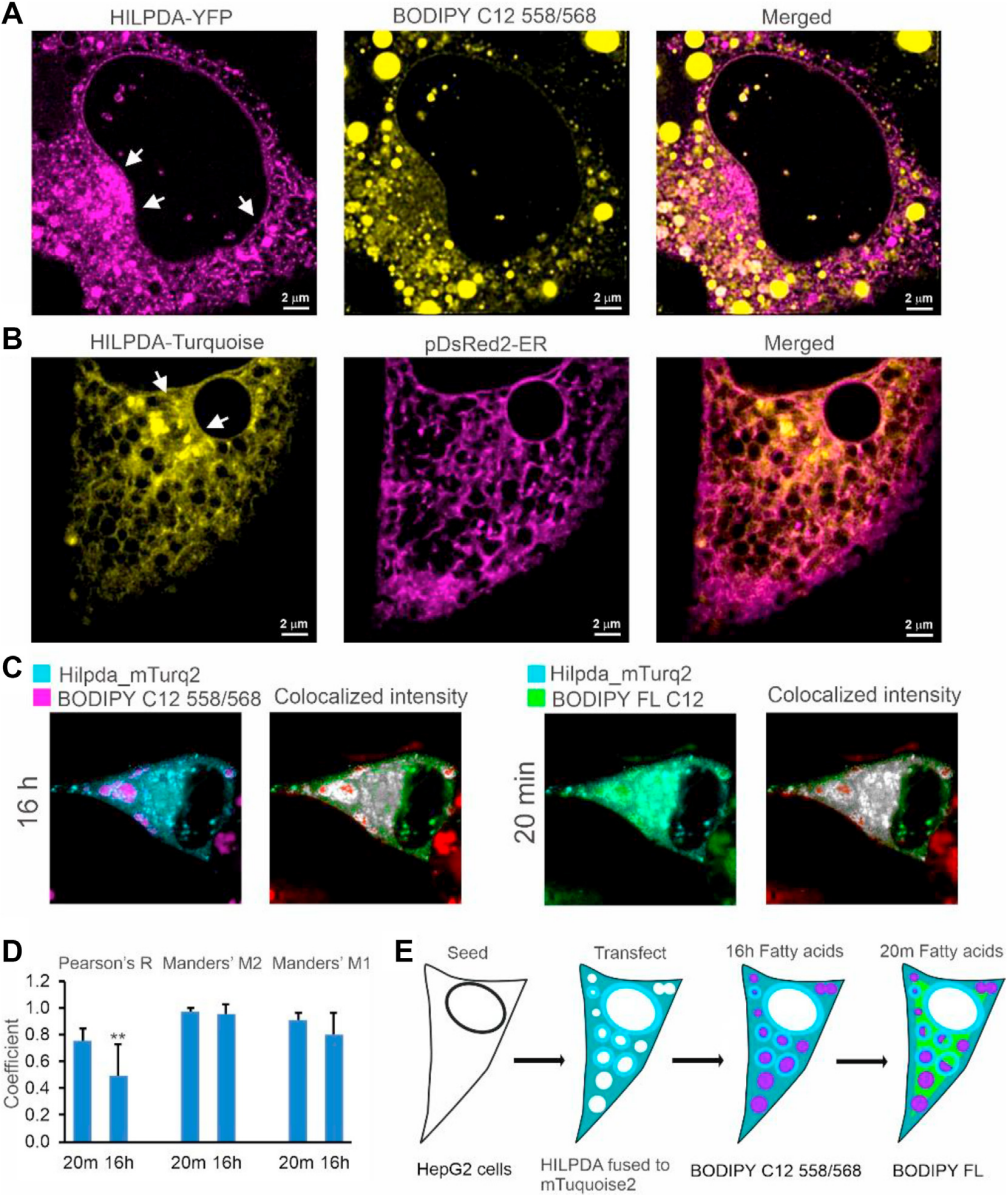
HILPDA is primarily localized to the perinuclear area and preferentially associated with new fat. A) STED microscopy of HepG2 cells transfected with HILPDA fused to sYFP2 and lipid loaded with 0.8 mM oleate and 15 μM BODIPY C12 558/568 for 18 h λ_ex_: 470 nm (YFP) and 558 nm (BODIPY-558/568). λ_em_: 480–540 nm (sYFP2) and 570–650 nm (BODIPY 558/568). Left panel: HILPDA-sYFP2; middle panel: BODIPY-558, right panel: overlay. Arrows indicate perinuclear area. B) HepG2 cells cotransfected with HILPDA fused to Turquoise2 and with ER marker pDsRed2-ER followed by incubation with 0.8 mM oleate overnight. Left panel: HILPDA-Turquoise2; middle panel: ER marker pDsRed2-ER, right panel: overlay. Arrows indicate perinuclear area. C) Confocal microscopy of HepG2 cells transfected with HILPDA fused to Turquoise2 and lipid loaded with 0.6 mM oleate and 15 μM BODIPY C12 558/568 for 16h, followed by incubation for 20 min with BODIPY FL C12 and fixed with 3.7% PFA. λ_ex_: 440 nm (mTurquoise2), 561 nm (BODIPY 558/568), and 488 nm (BODIPY FL). λ_em_: 450–480 nm (mTurquoise2), 570–620 nm (BODIPY 558/568), and 505–558 nm (BODIPY FL). Colocalized pixels of HILPDA and fluorescent fatty acids are represented on gray scale, higher colocalization is depicted with lighter pixels; non-colocalized HILPDA pixels are colored green; whereas non-colocalized fluorescent fatty acid pixels are colored red. D) Mean Pearson correlation coefficient R, and mean Manders' colocalization coefficients M2 and M1 (pixel by pixel analysis, n = 10 cells per group). Asterisk indicates significantly different according to Student's t-test; P < 0.01. E) Schematic depiction of the set-up and outcomes of the above experiments. Bar graphs and images are representative of multiple independent experiments.

To better focus on the intracellular localization of HILPDA, we used stimulated emission depletion (STED) microscopy, which generates images of very high spatial resolution (±70 nm). HepG2 cells were transfected with HILPDA fused to sYFP2, and the LD were visualized by lipid loading the cells with a mix of oleate and BODIPY C12 558/568. Interestingly, while some HILPDA was observed around LD, most of the HILPDA was localized in the perinuclear area, presumably representing the ER, where triglyceride synthesis occurs (Figure 4A). Interestingly, many LD were not surrounded by HILPDA. Visualization of the ER via co-transfection with pDsRed2-ER verified the partial localization of HILPDA in the ER (Figure 4B).

To examine the dynamics of the association of HILPDA with LD, we performed time-lapse fluorescence imaging in HepG2 cells transfected with HILPDA-sYFP2 and incubated with BODIPY FL C12 (Supplemental Video 1). Intriguingly, HILPDA was mainly present around LD that are being lipolyzed (disappearing) and remodeled (forming new LD). Little to no HILPDA was observed around stable LD. These data suggest a role of HILPDA in LD remodeling in liver cells.

Supplementary video related to this article can be found at https://doi.org/10.1016/j.molmet.2021.101168

The following is/are the supplementary data related to this article:

Multimedia component 2 Multimedia component 2

To better characterize the functional role of HILPDA in LD homeostasis in hepatocytes, we transfected HepG2 cells with HILPDA fused to mTurquoise2 and treated the cells with two labeled fatty acids that could be visualized separately using different channels. One labeled fatty acid (BODIPY C12 558/568) was added for 16 h, while the other labeled fatty acid (BODIPY FL C12) was added for 20 min, after which cells were fixed (Figure 4C). Colocalization was evaluated by Manders' colocalization coefficients and Pearson correlation coefficient (Figure 4D). Manders’ colocalization coefficients are measurements of co-occurrence, which is the spatial overlap of two probes. The Pearson correlation coefficient is a measurement of correlation, which evaluates the spatial overlap and signal proportionality. Analysis of the confocal images showed that HILPDA colocalizes almost entirely with the old and newly added fatty acids (M2: 96–97% respectively), and that the proportion of fatty acids that colocalized with HILPDA is greater for the newly added fatty acids than for the fatty acids added the day before (new M1:91% vs. old M1:80%). In line with this, the Pearson correlation coefficient was significantly higher for the newly added fatty acids than for the fatty acids added the day before (Figure 4D). These data indicate that HILPDA more strongly correlates with newly added fatty acids, which — assuming that most of the added fatty acids are converted into triglycerides — suggests that HILPDA preferentially colocalizes with newly synthesized triglycerides. A schematic depiction of the set-up and outcomes of the above experiments is presented in Figure 4E.

### 3.5 HILPDA increases DGAT activity and DGAT1 levels

**Figure 5:**
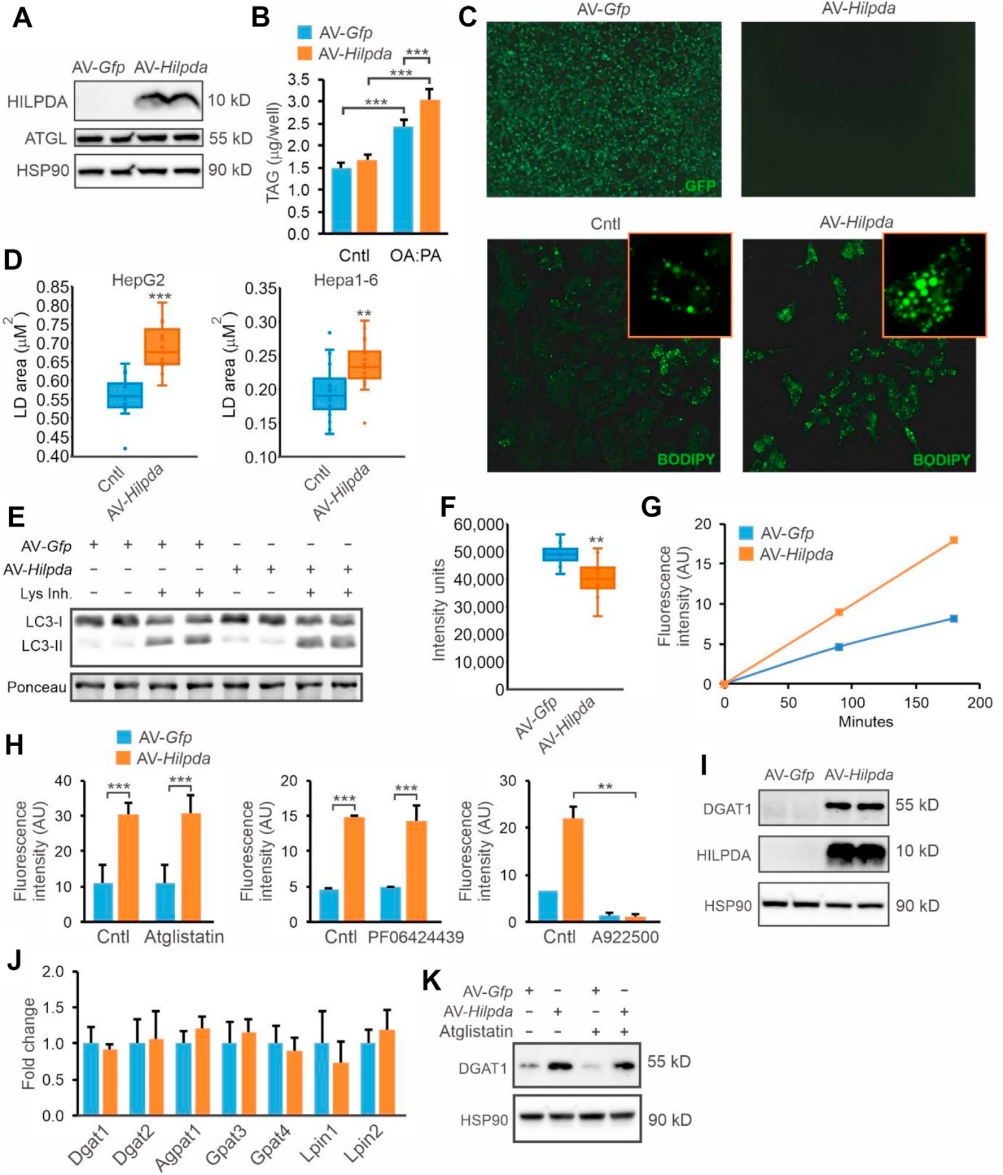
HILPDA overexpression promotes LD storage and increases DGAT1 levels in HepG2 cells. HepG2 cells were transduced with AV-*Hilpda*, AV-*Gfp* or non-transduced and treated with oleate:palmitate (2:1 ratio). A) HILPDA protein levels. B) Triglyceride content in HepG2 cells incubated with serum free DMEM for 3 h in 1 mM of oleate:palmitate. C) GFP fluorescence and BODIPY 493/503 staining. D) Quantification of LD size in HepG2 treated with 0.8 mM of oleate:palmitate for 8 h and Hepa 1–6 cells treated with 1 mM for 24 h. The graphs are representative of two independent experiments. E) LC3-I and LC3-II protein levels in HepG2 cells lipid loaded with 0.8 mM oleate:palmitate for 8 h, in the presence and absence of lysosomal inhibitor cocktail. F) Total DAG levels as determined by lipidomics in HepG2 cells incubated with 0.8 mM oleate:palmitate for 5h. G) Time course of DGAT activity in HepG2 cells. The graph is representative of three independent experiments. H) DGAT activity in HepG2 cells in the presence and absence of ATGL, DGAT2 and DGAT1 inhibitor. The graphs are representative of two independent experiments. I) DGAT1 and HILPDA protein levels in HepG2 cells. J) mRNA levels of selected genes. K) DGAT1 protein levels in HepG2 cells in presence and absence of Atglistatin. Asterisk indicates significantly different according to Student's t-test; ∗∗P < 0.01; ∗∗∗P < 0.001.

To further study how HILPDA promotes lipid storage, we overexpressed HILPDA in HepG2 cells via transduction with an adenoviral vector expressing *Hilpda.* HILPDA overexpression effectively raised HILPDA protein levels (Figure 5A) and was associated with a significant increase in triglyceride levels (Figure 5B) as well as LD size and abundance, as visualized by BODIPY staining (Figure 5C). Quantitative analysis showed that AV-*Hilpda* significantly increased the volume of the LD in both HepG2 and Hepa1-6 cells (Figure 5D). These data indicate that HILPDA overexpression promotes lipid storage in HepG2 cells.

Based on the finding that HILPDA increases lipid accumulation partly independently of ATGL, we considered the possibility that HILPDA may target the lipophagy pathway [32]. However, accumulation of the autophagosome marker LC3-II in the presence of lysosomal inhibitors was comparable between control and AV-*Hilpda* cells, indicating that increased LD content in AV-*Hilpda* cells is not due to stimulation of lipophagy (Figure 5E). Alternatively, we considered that HILPDA may promote the synthesis and/or storage of triglycerides. Lipidomics indicated that AV-*Hilpda* significantly decreased levels of diacylglycerols (Figure 5F) but did not significantly affect levels of other major lipid species (data not shown). Accordingly, we hypothesized that HILPDA might stimulate the activity of diacylglycerol acyltransferase (DGAT), which catalyzes the last and purportedly the rate-limiting step in the formation of triglycerides, using diacylglycerol and acyl-CoA as substrates. To determine the possible stimulatory effect of HILPDA on DGAT activity, we measured the synthesis of fluorescently-labeled triglycerides from fluorescent NBD-palmitoyl-CoA and 1,2 dioleoyl-sn-glycerol in lysates of HepG2 cells transduced with AV-*Hilpda* or AV-*Gfp*. Strikingly, the DGAT-mediated incorporation of fluorescent NBD-palmitoyl-CoA into triglycerides, as determined by quantification of thin-layer chromatography (TLC) plates, was markedly increased by HILPDA overexpression (Figure 5G). This increase in triglyceride synthesis in HepG2 cells was unaltered in the presence of Atglistatin, suggesting it is independent of ATGL (Figure 5H). DGAT activity is catalyzed by two different isozymes: DGAT1 and DGAT2 [33]. Whereas DGAT2 has a preference for endogenously synthesized fatty acids, DGAT1 mainly esterifies exogenous fatty acids to diacylglycerol [[34], [35], [36]]. Strikingly, the increase in triglyceride synthesis by HILPDA overexpression was unaltered by the DGAT2 inhibitor PF-06424439 but completely suppressed by the DGAT1 inhibitor A922500. The induction of DGAT activity in HepG2 cells by HILPDA overexpression was accompanied by a significant increase in DGAT1 protein levels (Figure 5I). The increase in DGAT1 protein was not associated with any change in mRNA levels of DGAT1, DGAT2, or other relevant proteins (Figure 5J), and was independent of ATGL activity (Figure 5K). The specificity of the DGAT1 antibody is shown in Supplemental Fig. 2a. Neither the DGAT1 inhibitor nor the DGAT2 inhibitor affected endogenous HILPDA levels, as tested in Hepa1-6 cells (Supplemental Figs. 2b and 2c).

**Figure 6:**
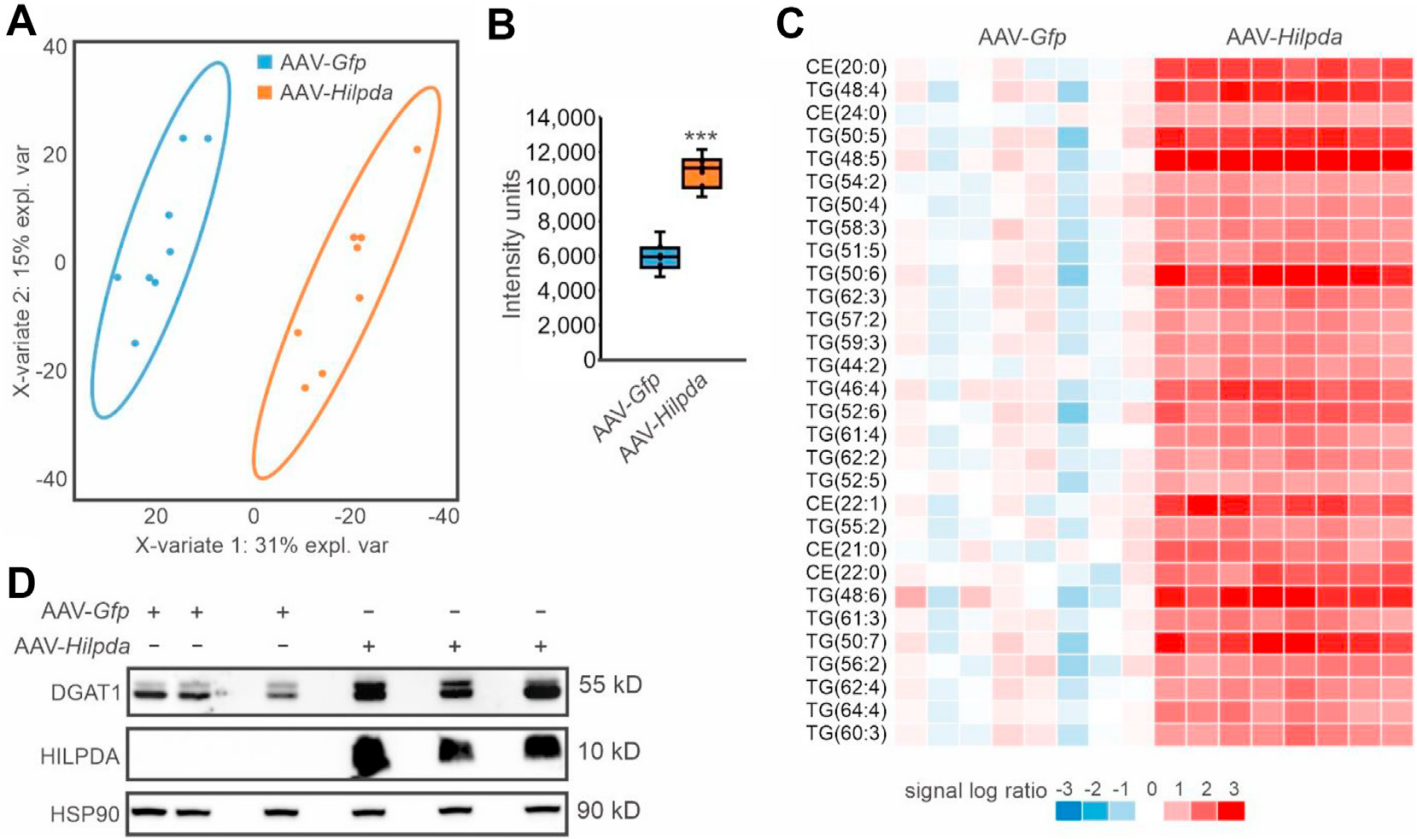
HILPDA overexpression promotes triglycerides storage and increases DGAT1 levels in the mouse liver. Livers were collected from mice 4 weeks after injection with AAV-*Gfp* or AAV-*Hilpda* [17]. A) PLS-DA analysis of the liver lipidomics profiles. B) Cumulative hepatic concentration of all triglyceride species. C) Heatmap of the 30 most significantly altered lipid species. D) DGAT1 and HILPDA protein levels in livers of mice infected with AAV-*Gfp* or AAV-*Hilpda*. N = 8 mice/group. Asterisk indicates significantly different according to Student's t-test; ∗∗∗P < 0.001.

To assess the effect of HILPDA overexpression on hepatic lipid levels and DGAT1 protein levels in vivo, we used samples from a previous study in which mice were infected with AAV expressing HILPDA [17]. AAV-mediated HILPDA overexpression markedly changed the lipidomic profile in the liver (Figure 6A) and markedly increased hepatic triglyceride levels (Figure 6B). In fact, out of 1,479 lipids measured, the 30 most significantly altered lipids by HILPDA overexpression were nearly all triglycerides, the remainder being cholesteryl-esters (Figure 6C). Consistent with the data in HepG2 cells, HILPDA overexpression increased DGAT1 protein levels in the mouse liver (Figure 6D). These data suggest that HILPDA promotes triglyceride storage in the liver concurrent with an increase in DGAT1 protein.

### 3.6 HILPDA physically interacts with DGAT1

**Figure 7:**
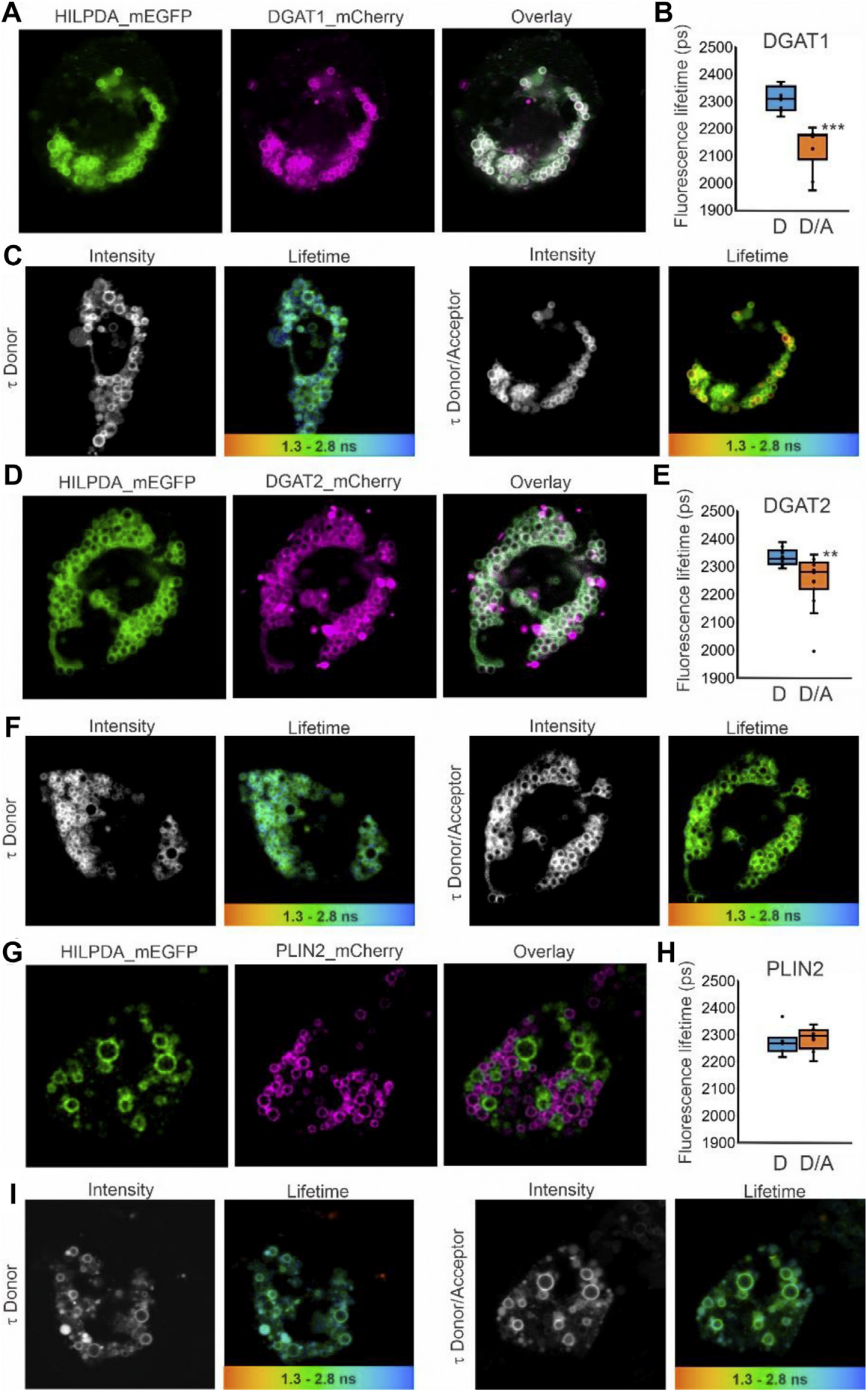
HILPDA and DGAT1/DGAT2 colocalize and physically interact intracellularly. HepG2 cells were transfected with HILPDA_mEGFP and DGAT1_mCherry or DGAT2_mCherry under lipid loaded conditions. Microscopy was carried out on live cells. A) HILPDA_EGFP and mDGAT1_mCherry partially colocalize in HepG2 cells. B) Fluorescence lifetime (τ) of HILPDA_EGFP in absence and presence of acceptor DGAT1_mCherry (n = 8 per condition). C) Intensity image and LUT colored lifetime image from red (1,300 ps) to blue (2,800 ps) from HILPDA_EGFP lifetime (τ) in the absence (left) or presence (right) of DGAT1_mCherry indicating where interaction occurs. D) HILPDA_EGFP and DGAT2_mCherry partially colocalize in HepG2 cells. E) Fluorescence lifetime (τ) of HILPDA_EGFP in the absence and presence of acceptor DGAT2_mCherry (n = 12–14). F) Intensity image and LUT colored lifetime image from red (1,300 ps) to blue (2,800 ps) from HILPDA_EGFP lifetime (τ) in the absence (left) or presence (right) of DGAT2_mCherry indicating where interaction occurs g) HILPDA_EGFP and PLIN2_mCherry do not colocalize in HepG2 cells. H) Fluorescence lifetime (τ) of HILPDA_EGFP in the absence and presence of acceptor PLIN2_mCherry (n = 5–6). Asterisk indicates significantly different from donor-only according to Student's t-test; ∗∗P < 0.01; ∗∗∗P < 0.001. D = donor, D/A = donor/acceptor. Graphs and images are representative of at least two independent experiments in live and fixed cells.

To investigate whether HILPDA may physically interact with DGAT1 in cells, we performed FRET quantified by FLIM. In live HepG2 cells transfected with HILPDA-mEGFP and DGAT1-mCherry, HILPDA colocalized with DGAT1 (Figure 7A). Because confocal microscopy is diffraction limited to 250 nm, our colocalization results do not directly demonstrate that HILPDA and DGAT1 are physically interacting. To determine protein interactions, we performed FRET quantified by FLIM. The mean fluorescence lifetime of the donor fluorophore HILPDA-EGFP was significantly decreased by the presence of the acceptor fluorophore DGAT1-mCherry (Figure 7B–C). This result demonstrates that HILPDA and DGAT1 are in very close proximity, indicating a direct physical interaction between these two proteins. Transfection of HepG2 cells with HILPDA-mEGFP and DGAT2-mCherry showed that HILPDA also partially colocalizes with DGAT2 (Figure 7D). As for DGAT1, the mean fluorescence lifetime of the donor HILPDA-EGFP was significantly decreased upon co-transfection of the acceptor DGAT2-mCherry (Figure 7E–F). These data indicate that HILPDA is able to physically interact with both DGAT1 and DGAT2. By contrast, although GPAT4-EFGP and HILPDA-mCherry showed substantial colocalization, FRET-FLIM analysis did not reveal a significant change in donor lifetime, indicating that these proteins do not interact (Supplemental Figs. 3a–c). Also, no significant change in donor lifetime was observed for HILPDA-mEGFP in combination with PLIN3-mCherry or GPAT1-EGFP in combination with HILPDA-mCherry (Supplemental Fig. 3d). Furthermore, HILPDA-mEGFP showed little to no colocalization with PLIN2-mCherry (Figure 7G–H). The latter observation was confirmed in Hepa1-6 and 3T3-L1 cells (Supplemental Fig. 3e).

We repeated the FRET-FLIM experiments in fixed HepG2 cells and obtained similar outcomes. Specifically, co-expression of HILPDA-mEGFP with DGAT1-mCherry (Supplemental Figs. 4a–c) and DGAT2-mCherry (Supplemental Figs. 4d–f) led to a significant reduction in donor fluorescence lifetime. Collectively, these data indicate that HILPDA physically interacts with DGAT1 and DGAT2 but not with any of the other proteins studied.

**Figure 8:**
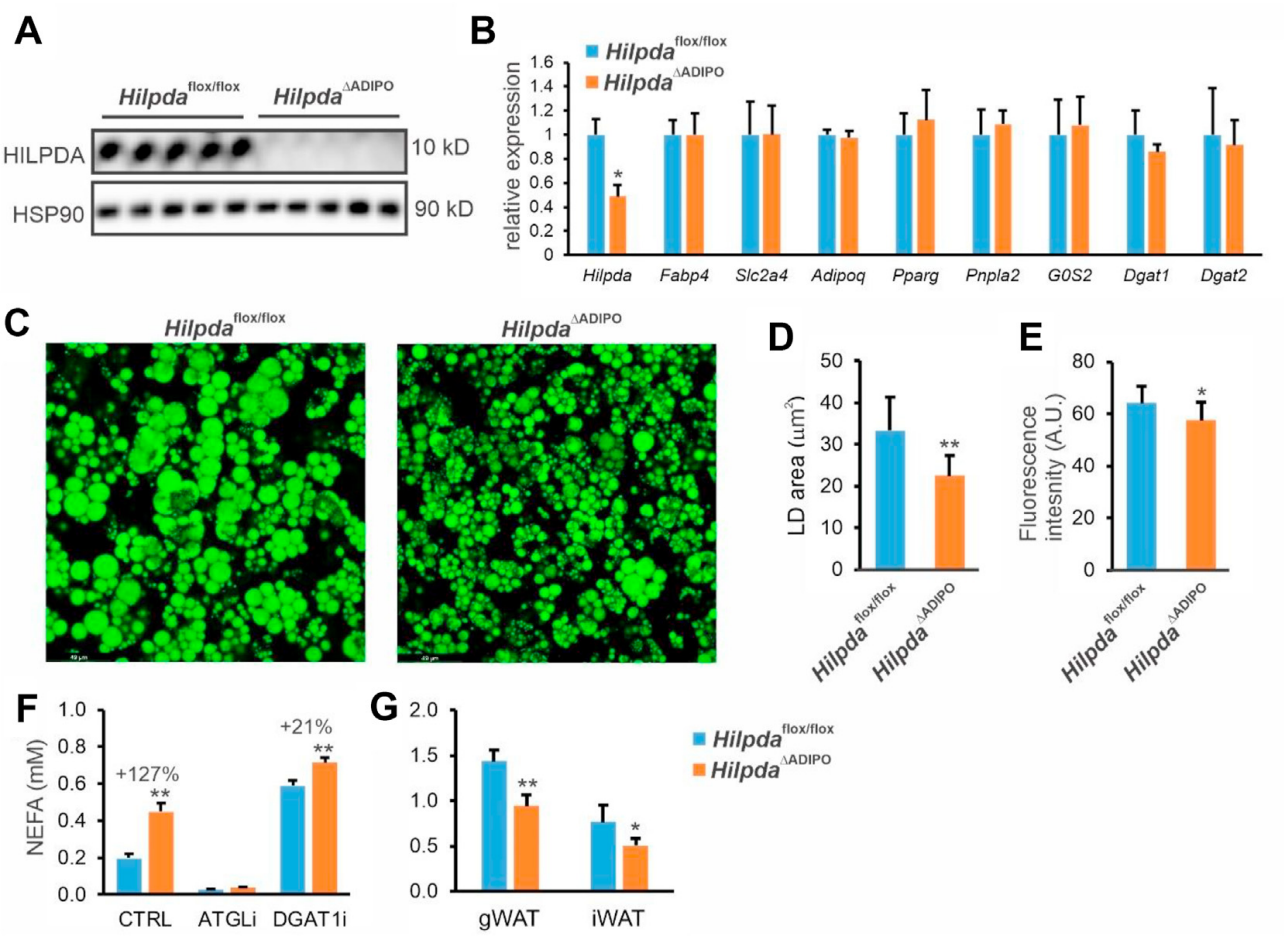
HILPDA promotes lipid storage and reduces fatty acid secretion in adipocytes via DGAT1. Primary adipocytes were differentiated using the adipose tissue of *Hilpda*^ΔADIPO^ and *Hilpda*^flox/flox^ mice. A) HILPDA protein levels as determined by Western blot. B) mRNA levels of selected genes determined by qPCR. C) Confocal fluorescence microscopy of Bodipy-stained *Hilpda*^ΔADIPO^ and *Hilpda*^flox/flox^ adipocytes. D) Quantification of the lipid droplet area. E) Quantification of the Bodipy fluorescence. F) Concentration of fatty acids in the medium of *Hilpda*^ΔADIPO^ and *Hilpda*^flox/flox^ adipocytes treated with Atglistatin (ATGLi) or T863 (DGAT1i). Graphs and images are representative of at least two independent experiments. G) Weight of the gonadal and inguinal fat depots in *Hilpda*^ΔADIPO^ and *Hilpda*^flox/flox^ mice. N = 11–13 mice/group. Asterisk indicates significantly different from *Hilpda*^flox/flox^ mice/cells according to Student's t-test; ∗P < 0.05; ∗∗P < 0.01.

Finally, to investigate whether HILPDA may promote lipid storage via DGAT in other cell types, we turned our attention to adipocytes. Adipocyte-specific HILPDA-deficient mice (*Hilpda*^ΔADIPO^) were generated by crossing *Hilpda*^flox/flox^ mice with mice expressing Cre-recombinase under control of the adiponectin promoter and used for the production of HILPDA-deficient primary adipocytes. HILPDA protein levels were markedly reduced in *Hilpda*^ΔADIPO^ adipocytes (Figure 8A). Deficiency of HILPDA in primary mouse adipocytes did not influence adipogenesis per se, as indicated by unaltered expression of adipogenic marker genes, nor did it influence the expression of *G0s2*, *Dgat1*, or *Dgat2* (Figure 8B). However, lipid accumulation was markedly reduced by HILPDA deficiency (Figure 8C), as revealed by a significant decrease in the size of the lipid droplets (Figure 8D) and the Bodipy fluorescence (Figure 8E). To determine the underlying mechanism, we measured the release of non-esterified fatty acids into the medium. Interestingly, HILPDA deficiency significantly increased fatty acid secretion (Figure 8F). Secretion of fatty acids was completely blunted by the ATGL inhibitor Atglistatin, indicating that fatty acid secretion is entirely driven by ATGL-mediated lipolysis. Theoretically, the increased fatty acid secretion in HILPDA-deficient adipocytes may be caused by enhanced lipolysis or reduced fatty acid re-esterification. As expected, blocking fatty acid (re)esterification by inhibiting DGAT1 increased fatty acid release. Interestingly, the relative increase in fatty acid secretion by HILPDA deficiency was markedly reduced upon DGAT1 inhibition (Figure 8F), suggesting that the elevated fatty acid secretion in HILPDA-deficient adipocytes cannot solely be explained by enhanced ATGL-mediated lipolysis but also involves impaired DGAT1-mediated fatty acid re-esterification. Finally, in agreement with a stimulatory effect of HILPDA on lipid storage, the weights of the epididymal and inguinal fat depots were significantly lower in *Hilpda*^ΔADIPO^ mice than *Hilpda*^flox/flox^ mice (Figure 8G).

## 4 Discussion

The purpose of this study was to better define the role and mechanism of action of HILPDA in liver cells. We found that HILPDA is induced by fatty acids in liver cells and stimulates lipid storage, which occurs at least partly independently of ATGL. Fluorescence microscopy showed that HILPDA partly colocalizes with LD and with the ER, is especially abundant in perinuclear areas, mainly associates with newly added fatty acids, and preferentially localizes to LD that are being remodeled. Mechanistically, HILPDA physically interacts with DGAT1, stimulates DGAT activity, and increases DGAT1 protein levels. Studies in adipocytes further support the stimulatory effect of HILPDA on lipid storage via DGAT1. Our data suggest that in addition to inhibiting ATGL-mediated lipolysis, HILPDA stimulates DGAT1-mediated triglyceride synthesis. Based on our and other data, it can be hypothesized that HILPDA is part of a larger triglyceride turnover complex (“lipolysome”) that includes enzymes involved in triglyceride synthesis and triglyceride breakdown, including ATGL and DGAT1, as well as regulatory proteins, such as ABHD5 and G0S2 [33,37,38].

HILPDA and G0S2 share extensive sequence homology, and both proteins are able to inhibit ATGL. Recently, evidence was provided that G0S2 not only suppresses lipolysis but also promotes triglyceride synthesis by carrying glycerol-3-phosphate acyltransferase (GPAT/LPAAT/AGPAT) enzymatic activity [39]. Given the very small size of HILPDA (63 amino acids), it is unlikely that HILPDA can itself function as a fatty acid esterification enzyme. Rather, our data suggest that HILPDA increases the level and activity of the DGAT1 enzyme via a direct physical interaction between the two proteins. DGAT1 catalyzes the esterification of exogenous fatty acids and fatty acids released from LD [35,40]. How HILPDA increases DGAT1 levels is unclear, but it can be hypothesized that the binding of HILPDA may stabilize DGAT1. Currently, nothing is known about how DGAT1 is degraded, complicating the study of the possible role of HILPDA in DGAT1 degradation.

PLIN2 is considered to represent a general lipid droplet-coating protein that occurs in essentially all cells, including hepatocytes. Evidence abounds indicating that PLIN2 promotes LD formation and thereby protects stored triglycerides from lipolysis [41]. Although PLIN2 is believed to reside on most LD in hepatocytes, we found that HILPDA-mEGFP did not colocalize with PLIN2-mCherry and that HILPDA and PLIN2 coat distinct sets of LD. Based on this observation, it can be hypothesized that the presence of HILDPA on LD may lead to the exclusion of PLIN2 or vice-versa. Whereas coating with PLIN2 may define more stable LD, coating with HILPDA may be a feature of LD that are being remodeled via active triglyceride synthesis and lipolysis. This possibility merits further experimental investigation.

In this paper, deficiency of HILPDA in the mouse liver led to a modest reduction in triglyceride storage after inducing NASH. A previous study found that HILPDA deficiency does not significantly influence hepatic triglyceride levels in mice fed a high-fat diet [19]. The reason for the divergent results is unclear but could be related to the different types of diets used. Although statistically significant, the magnitude of the effect of HILPDA deficiency on hepatic triglyceride levels in mice was modest, which may be explained by the relatively low expression of *Hilpda* in the mouse liver. By contrast, raising liver HILPDA levels by AAV markedly elevates triglyceride storage.

Whereas HILPDA deficiency only had a modest effect on triglyceride storage in the mouse liver, deficiency of HILPDA markedly reduced lipid storage in primary hepatocytes, which is consistent with the much higher *Hilpda* expression in primary mouse hepatocytes compared to the mouse liver. Specific physiological, pathological, and pharmacological stimuli may elevate HILPDA levels, thereby rendering HILPDA more important. A pathological condition associated with upregulation of *Hilpda* is infection with hepatitis C virus [42], which, interestingly, uses lipid droplets for replication [43,44]. Also, as HILPDA is highly induced by hypoxia and HIF1α, HILPDA is an excellent candidate to mediate the stimulatory effect of hypoxia/HIF1α on hepatic triglyceride levels [22,45]. We speculate that an elevated expression of HIF1α may also explain the higher expression of HILDPA in primary hepatocytes compared to the mouse liver.

The observed reduction in lipid storage in HILPDA-deficient hepatocytes is consistent with the data by DiStefano et al. [19]. According to their fatty acid flux data, the decrease in lipid storage is explained by a combination of decreased fatty acid uptake, increased fatty acid beta-oxidation, and increased triglyceride lipolysis. While triglyceride lipolysis is known to be directly targeted by HILPDA, it is unclear whether fatty acid uptake and β-oxidation are as well, or if they are affected indirectly.

Currently, little is known about HILPDA in human liver. If the expression level of *HILPDA* in human liver is sufficiently high, inactivation of HILPDA could in theory be a promising strategy to treat non-alcoholic fatty liver disease. Whether NAFLD is associated with a change in the expression of *HILPDA* in the human liver is unknown. Because loss-of-function variants in *HILPDA* would be expected to lead to reduced hepatic lipid storage, *HILPDA* is unlikely to emerge from any genome-wide association screens on NAFLD. Using multiple tools, we searched for SNP missense variants in the protein-coding region of the *HILPDA* gene. We identified several missense variants, several of which were predicted to have a negative impact on protein structure. However, all identified missense variants are rare or very rare with a minor allele frequency <0.1%. Accordingly, human genetic studies are unlikely to clarify the role of HILPDA in the human liver.

As our gene targeting strategy was directed towards HILPDA in hepatocytes, our conclusions are also limited to the role of HILPDA in these cells. The fact that albumin Cre-mediated *Hilpda* deletion only reduced hepatic *Hilpda* mRNA by approximately 50–60% suggests that *Hilpda* is expressed in other liver cell types as well, including possibly Kupffer cells, stellate cells, and endothelial cells. Given the important role of HILPDA in lipid storage in macrophages, it would be of interest to study the effect of LysM-Cre mediated HILPDA deficiency on NASH and on lipid storage in Kupffer cells.

In our study, expression of HILPDA in liver cells was induced by fatty acids, which is consistent with the very sensitive upregulation of HILPDA by fatty acids in macrophages [18,24]. In addition to activating *Hilpda* transcription via PPARs [17,24], it is possible that fatty acids also specifically upregulate HILPDA at the protein level. The marked upregulation of *Hilpda* by fatty acids is likely part of a feed-forward mechanism to properly dispose of the fatty acids by promoting their storage as triglycerides, either by activating the last step in triglyceride synthesis and/or inhibiting the first step in triglyceride breakdown, thereby avoiding potentially lipotoxic levels of fatty acids.

Our study has several limitations. First, studies in cell culture were performed with overexpressed and tagged proteins, which may have influenced the results. It should be noted, however, that the colocalization of HILPDA to the ER and lipid droplets is fully in agreement with previous immunofluorescence studies on endogenous HILPDA [24,46]. Moreover, to study protein–protein interactions in live cells via FRET-FLIM, it is necessary to overexpress and tag proteins. Second, the expression of *Hilpda* in the mouse liver is low, certainly compared to macrophages, limiting the impact of HILPDA deficiency. Nevertheless, we could clearly detect HILPDA protein by Western blot in the mouse liver and observed a marked decrease in HILPDA abundance in hepatocyte-specific HILPDA-deficient mice. Third, direct evidence showing that physiological levels of HILPDA regulate triglyceride storage in the mouse liver via DGAT1 is lacking. Addressing this question is extremely challenging. Instead, we showed that the stimulatory effect of HILPDA overexpression on triglyceride synthesis in liver cells is mediated by DGAT1. Furthermore, we show in adipocytes that the inhibitory effect of HILPDA on fatty acid release is dependent on DGAT1. Fourth, the only direct evidence for a physical interaction between HILPDA and DGAT1 is via FRET-FLIM. Unfortunately, numerous efforts to immunoprecipitate HILPDA in cells failed, which is common for LD-associated proteins.

In conclusion, HILPDA serves as an intracellular sensor for fatty acids that couples changes in intracellular fatty acid levels to lipid storage. Specifically, our data suggest that in addition to inhibiting ATGL-mediated lipolysis, HILPDA increases lipid storage in cells by stimulating DGAT1-catalyzed triglyceride synthesis.
