# Supplementary material for: Hypoxia-inducible lipid droplet-associated induces DGAT1 and promotes lipid storage in hepatocytes
Source: Mol Metab. 2021 Jan 16;47:101168. doi: 10.1016/j.molmet.2021.101168 (PMC7881268; doi:10.1016/j.molmet.2021.101168)
Supplement: Multimedia component 1 [file mmc1.pdf]

SUPPLEMENTAL MATERIAL

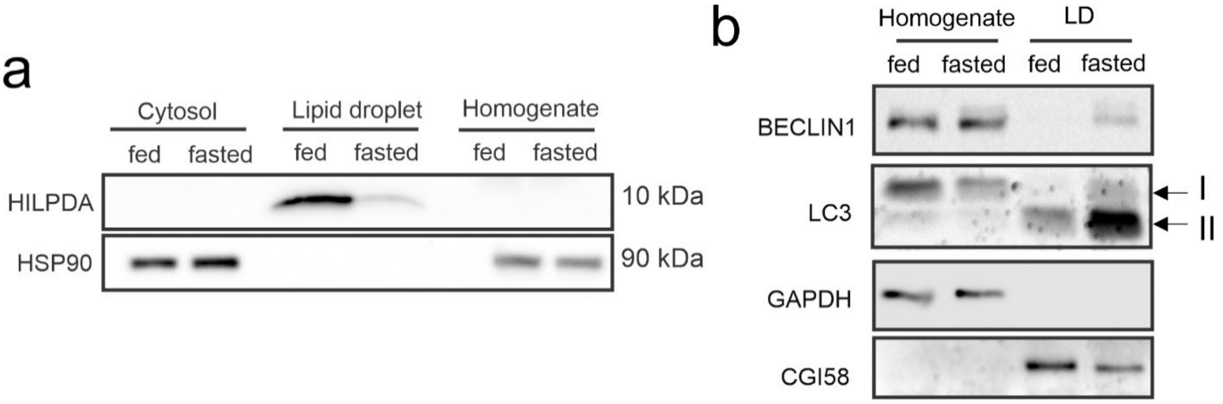

Supplemental figure 1. a) Enrichment of HILPDA in the lipid droplet fraction of fasted and fed wildtype livers as shown by immunoblot. b) Immunoblots showing the presence of autophagy-related proteins Beclin 1 and LC in homogenate and lipid droplets fractions of livers of fed and 24h fasted mice. Five livers of wildtype C57Bl/6 mice were pooled into each sample. Absence of GAPDH validates absence of cytosolic contamination of lipid droplet fractions. Presence of CGI58 (ABHD5) demonstrates enrichment of the LD fraction.

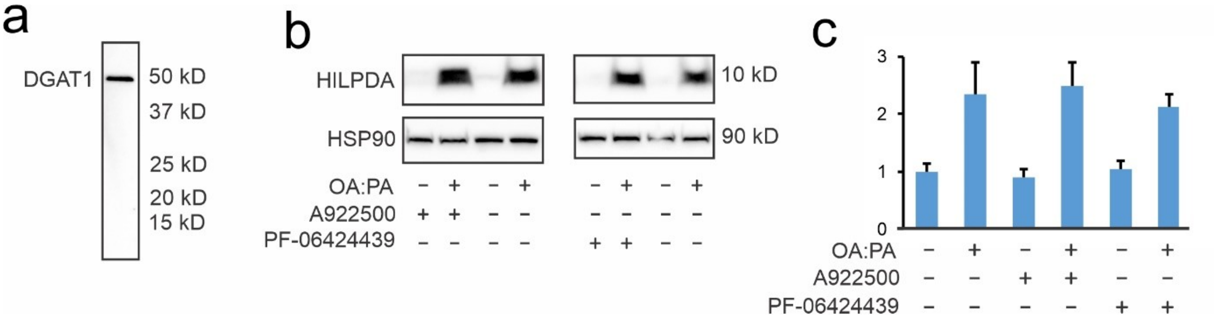

Supplemental figure 2. a) The DGAT1 antibody effectively detects DGAT1 in HepG2 cells transfected with DGAT1 expression vector. No effect of DGAT1 or DGAT2 inhibition on HILPDA protein levels (b) or mRNA levels (c) in Hepa1-6 cells. Hepa1-6 cells were pre-treated with DGAT1 inhibitor (A922500, 1 µM) or DGAT2 inhibitor (PF-06424439, 20 µM) for 30 minutes followed by co-treated for 24 hours with a 2:1 mixture of oleate and palmitate (total concentration (0.6 mM). Error bars represent SD.

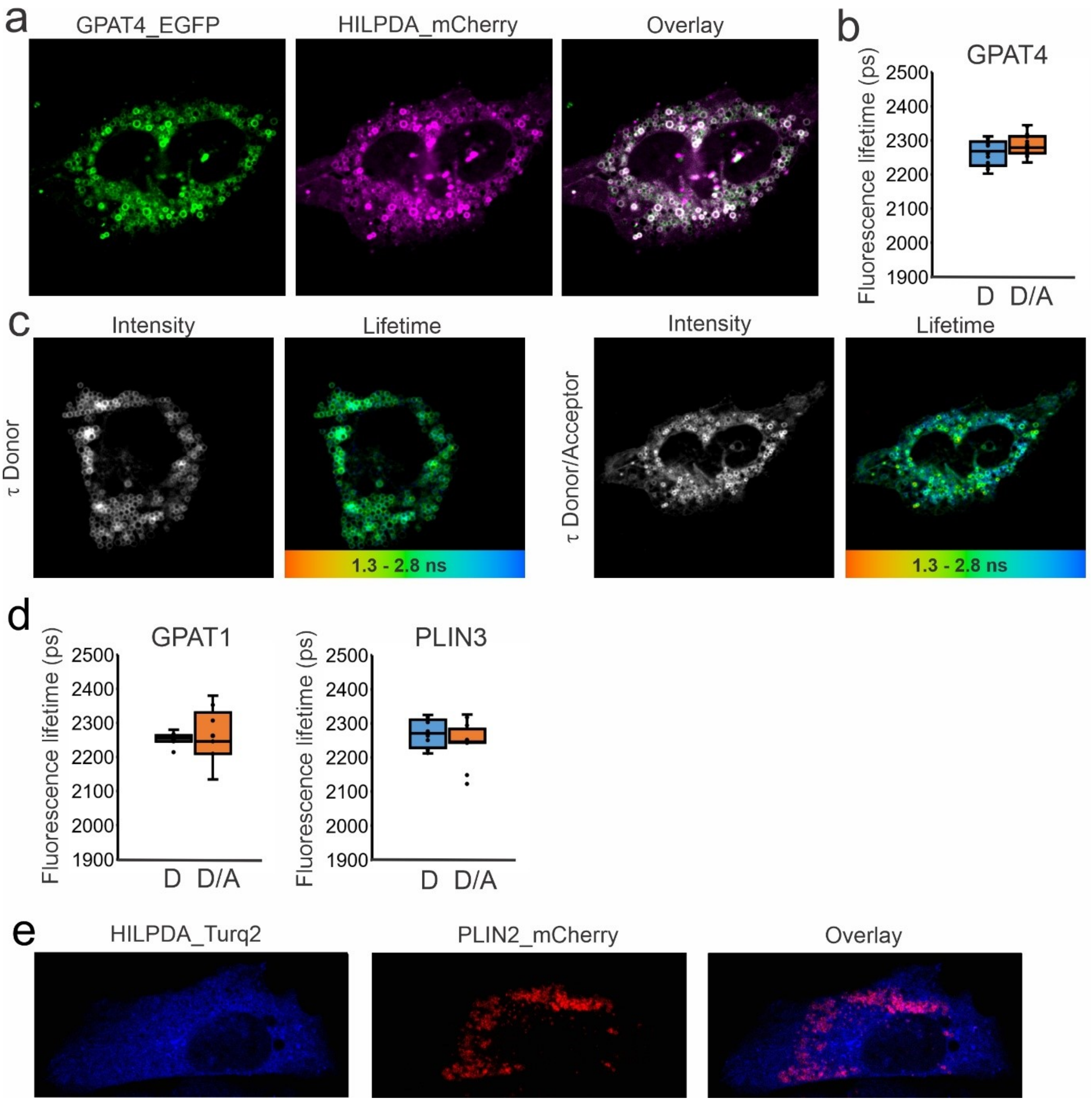

Supplemental figure 3. HepG2 cells were transfected with HILPDA_mCherry and GPAT4_EGFP, GPAT1_EGFP or HILPDA_EGFP and PLIN3_mCherry under lipid loaded conditions. Microscopy was carried out on live cells. a) HILPDA_mCherry and GPAT4_EGFP colocalize in HepG2 cells. b) Fluorescence lifetime (τ) of GPAT4_EGFP in absence and presence of acceptor HILPDA_mCherry. c) Intensity image and LUT coloured lifetime image from GPAT4_EGFP lifetime (τ) in the absence (left) or presence (right) of HILPDA_mCherry. d) Fluorescence lifetime (τ) of GPAT1_EGFP in absence and presence of acceptor HILPDA_EGFP and fluorescence lifetime (τ) of donor HILPDA_EGFP in the absence and presence of acceptor PLIN3_mCherry. e) 3T3-L1 cells were transfected with PLIN2_mCherry and HILPDA_Turq2 and incubated overnight with 0.025% Tween 80 for LD formation. Images were acquired sequentially. λex: 543 nm (mCherry) and 458 nm (Turquoise2). λem: 560-615 nm (mCherry) and 470-500 nm (Turquoise2).

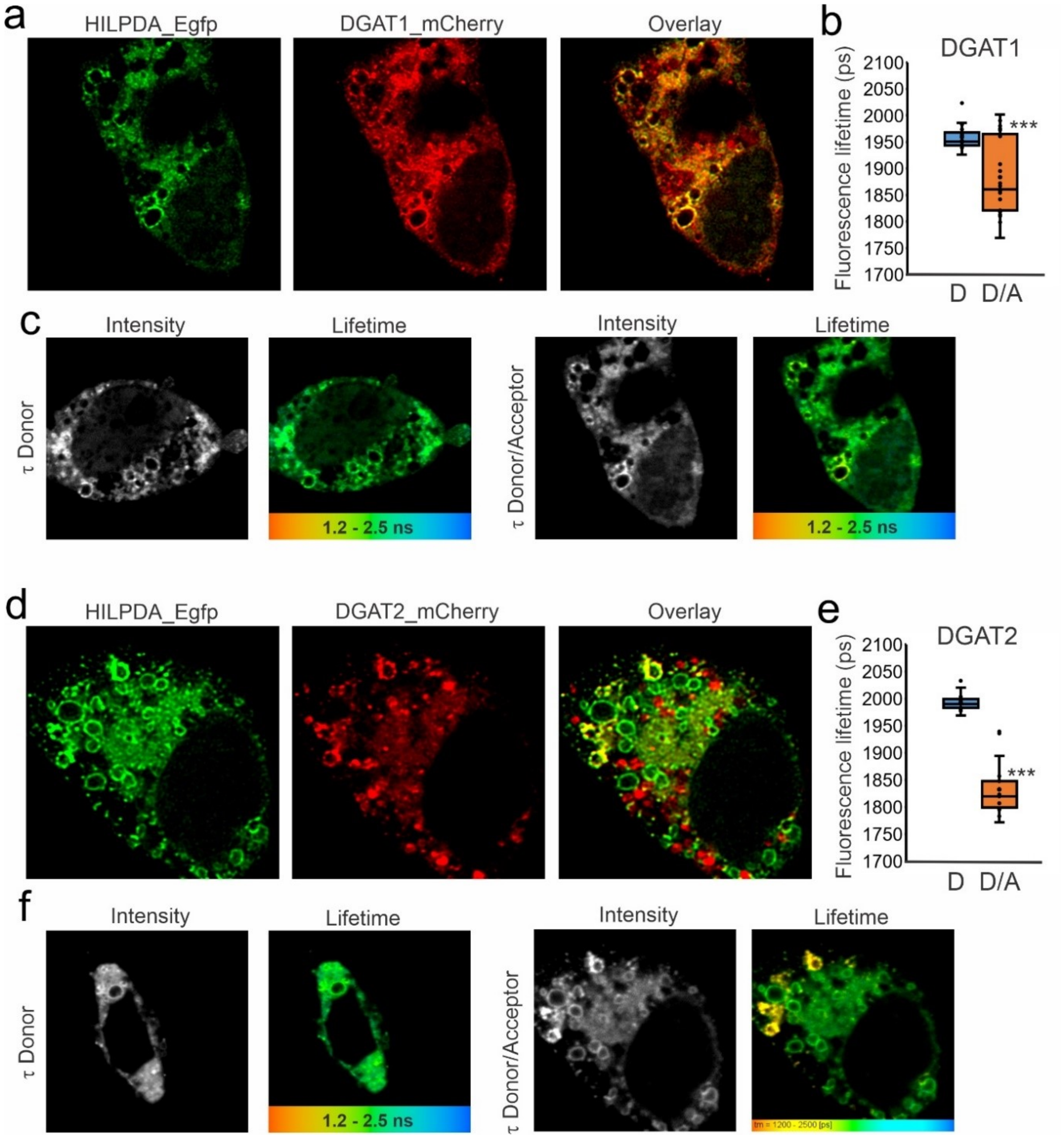

Supplemental figure 4. HILPDA and DGAT1/DGAT2 colocalize and physically interact intracellularly. HepG2 cells were transfected with HILPDA_EGFP and DGAT1_mCherry or DGAT2_mCherry under lipid loaded conditions. Microscopy was carried out on fixed cells. a) HILPDA_EGFP and mDGAT1_mCherry partially colocalize in HepG2 cells. b) Fluorescence lifetime (τ) of HILPDA_EGFP in absence and presence of acceptor DGAT1_mCherry. c) Intensity image and LUT coloured lifetime image from red (1200 ps) to blue (2500 ps) from HILPDA_EGFP lifetime (τ) in the absence (left) or presence (right) of DGAT1_mCherry indicating where interaction occurs) HILPDA_EGFP and DGAT2_mCherry partially colocalize in HepG2 cells. e) Fluorescence lifetime (τ) of HILPDA_EGFP in absence and presence of acceptor DGAT2_mCherry. f) Intensity image and LUT coloured lifetime image from red (1200 ps) to blue (2500 ps) from HILPDA_EGFP lifetime (τ) in the absence (left) or presence (right) of DGAT2_mCherry indicating where interaction occurs. Asterisk indicates significantly different from donor only according to Student's t test; ***$P < 0.001$.

Legend to Video 1:

HILPDA concentrates in active (lipolyzed/re-emerged) lipid droplets. HepG2 cells were transfected with HILPDA fused to mCherry, the day after transfection cells were starved for 1h with HBSS 0.2% FA-free BSA. Medium was then replaced with QBT fatty acid uptake assay kit, which contains a BODIPY dodecanoic acid fluorescent fatty acid and after 4h incubation cells were imaged on a Leica TCS SP8 X system. λ^{ex}: 561 nm (mCherry) and 488 nm (BODIPY). λ^{em}: 570-620 nm (mCherry) and 505-550 nm (BODIPY). Images were acquired sequentially using 512 x 512. Left, Hilpda in magenta LUTs and fatty acid BODIPY in green LUTs. White tone depicts higher signal intensity from HILPDA_mCherry. Right, Hilpda_mCherry distribution is delineated in white and the underneath fatty acid BODIPY can be visualized in green LUTs.
